# Monolithic Perovskite/Perovskite/Silicon Triple-Junction Solar Cells: Fundamentals, Progress, and Prospects

**DOI:** 10.1007/s40820-025-01836-8

**Published:** 2025-07-21

**Authors:** Leiping Duan, Xin Cui, Cheng Xu, Zhong Chen, Jianghui Zheng

**Affiliations:** 1 School of Electronic Science and Engineering, Xiamen University, Xiamen, 361005 Fujian People’s Republic of China; 2 Hiking PV Technology Co. Ltd., Shenzhen, 518109 Guangdong People’s Republic of China; 3 Research Institute of Renewable Energy and Advanced Materials, Zijin Mining Group Co. Ltd, Xiamen, 361101 Fujian People’s Republic of China; 4 Shenzhen Research Institute of Xiamen University, Shenzhen, 518057 Guangdong People’s Republic of China

**Keywords:** Tandem solar cell, Perovskite, Triple-junciton solar cell, Photovoltaic

## Abstract

Crystalline silicon (c-Si) solar cells, though dominating the photovoltaic market, are nearing their theoretical power conversion efficiencies (PCE) limit of 29.4%, necessitating the adoption of multi-junction technology to achieve higher performance. Among these, perovskite-on-silicon-based multi-junction solar cells have emerged as a promising alternative, where the perovskite offering tunable bandgaps, superior optoelectronic properties, and cost-effective manufacturing. Recent announced double-junction solar cells (PSDJSCs) have achieved the PCE of 34.85%, surpassing all other double-junction technologies. Encouragingly, the rapid advancements in PSDJSCs have spurred increased research interest in perovskite/perovskite/silicon triple-junction solar cells (PSTJSCs) in 2024. This triple-junction solar cell configuration demonstrates immense potential due to their optimum balance between achieving a high PCE limit and managing device complexity. This review provides a comprehensive analysis of PSTJSCs, covering fundamental principles, and technological milestones. Current challenges, including current mismatch, open-circuit voltage deficits, phase segregation, and stability issues, and their corresponding strategies are also discussed, alongside future directions to achieve long-term stability and high PCE. This work aims to advance the understanding of the development in PSTJSCs, paving the way for their practical implementation.

## Highlights

- Perovskite/perovskite/silicon triple-junction solar cells (PSTJSCs) are emerging as a promising strategy to exceed the efficiency limits of traditional silicon solar cells.
- This review systematically analyses the key principles, recent breakthroughs, and remaining challenges in PSTJSC development, including current mismatch, open-circuit voltage loss, phase segregation, and stability.
- Strategies to address these issues and future directions toward achieving high efficiency and long-term operational stability are comprehensively discussed.

## Graphical abstract

**Figure:**
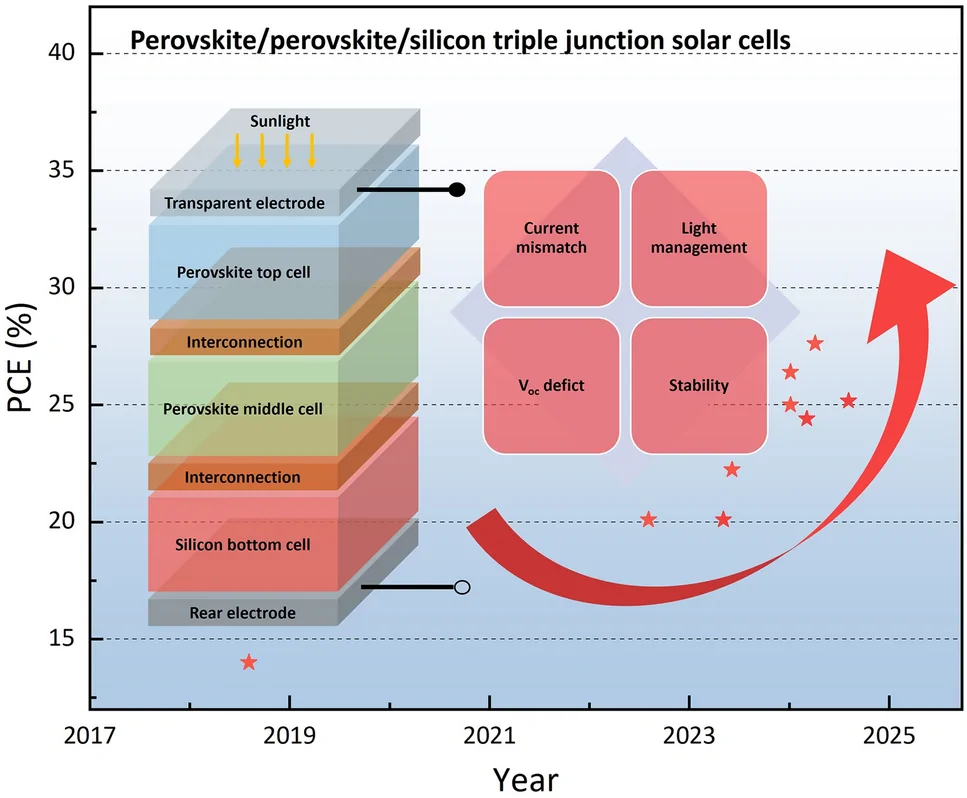

## Introduction

In response to global decarbonization policies, the development of next-generation sustainable energy technologies has become critical [1, 2]. Among these, photovoltaics (PV) is a key technology, providing clean energy solutions to address the growing demands of an increasingly resource-intensive and fast-evolving world [3]. Over the past decade, the manufacturing costs of mainstream PV modules have significantly decreased, now accounting for less than half of the total cost of utility-scale solar PV installations [4]. In contrast, non-module costs, commonly known as balance of system (BOS) costs, scale with the area of deployed PV rather than the power generated. Therefore, enhancing the power conversion efficiency (PCE) of solar cells to increase the power output per unit area emerges as the most effective strategy for further reducing the overall cost of PV-generated electricity [5, 6]. To date, crystalline silicon (c-Si) solar cells have dominated over 97% of the PV market, driving significant large-scale deployment [4]. The record PCE of c-Si solar cells has reached 27.3% [7], approaching the theoretical maximum PCE of 29.4% described by the Shockley–Queisser (SQ) limit for single-junction c-Si technology [8, 9], thereby offering limited headroom for further enhancements.

**Fig. 1:**
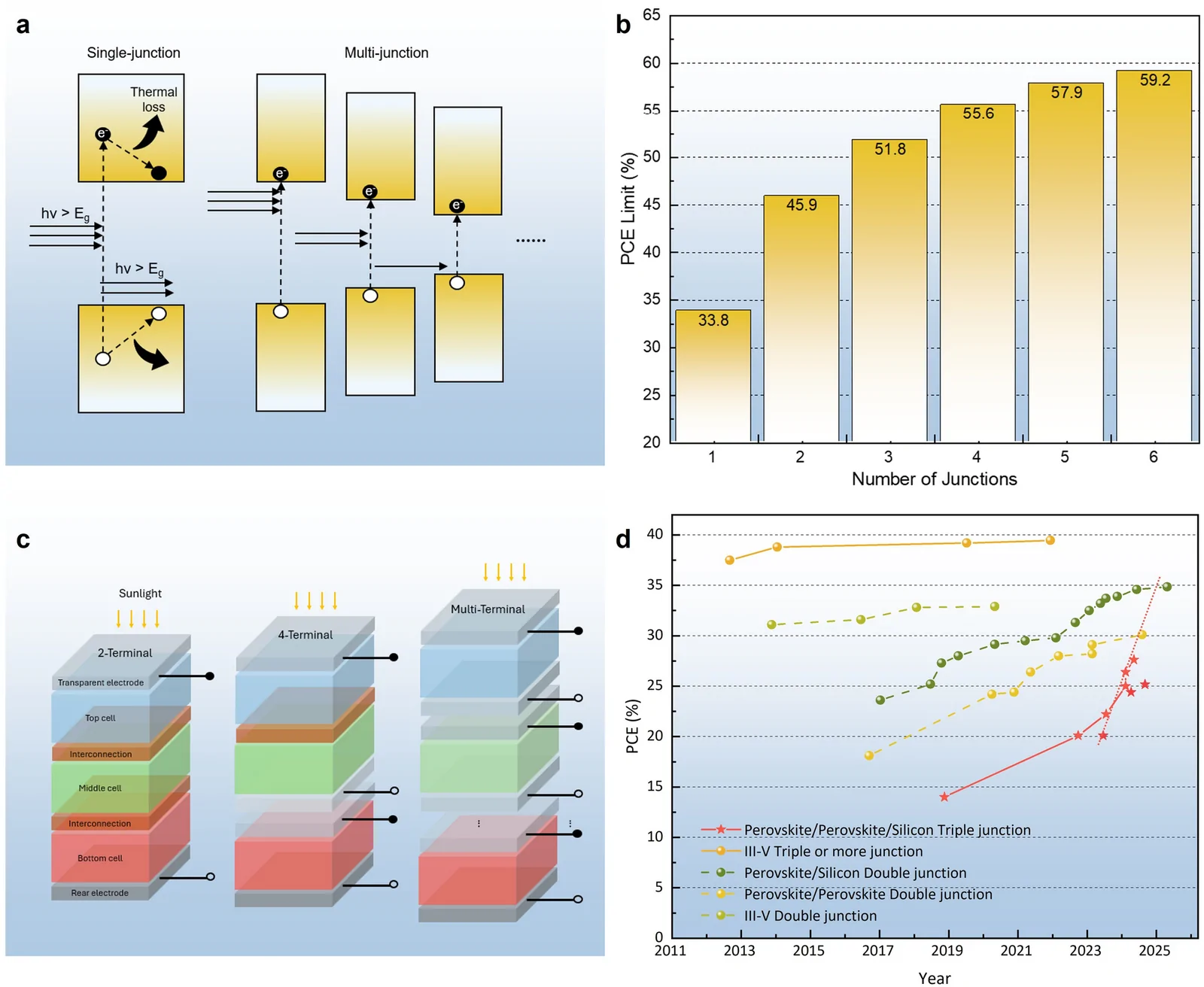
**a** Schematic illustration showing the light absorption in single and multi-junction solar cells. Reproduced with permission from Ref. [9]. Copyright 2017 Springer Nature. **b** The theoretical efficiency of multi-junction solar cells in the radiative limit is analyzed as a function of the number of junctions, with optimal bandgap combinations provided for achieving maximum PCE under the AM 1.5g spectrum. Reproduced with permission from Ref. [10]. Copyright 2024 The Royal Society of Chemistry. **c** Schematic diagram of two-terminal (monolithic), four-terminal, and multi-terminal designs in multi-junction solar cells. **d** PCE progression of double and triple-junction solar cells incorporating III-V, silicon, and perovskite in their architecture

One of the well-established methods for overcoming the SQ limit is multi-junction technology [11, 12]. In this approach, multiple solar cells with different bandgaps are stacked in descending order of bandgap energy, with the wide-bandgap cell positioned at the top, facing the sun [13]. As illustrated in Fig. 1a (right), the top cell absorbs high-energy photons up to its bandgap, while the lower-energy photons pass through and are absorbed by the subcells beneath, each with progressively narrower bandgaps [9]. Compared to single-junction as demonstrated in Fig. 1a (left), multi-junction configuration minimizes thermalization losses and enhances the overall capture of the solar spectrum [14]. As shown in Fig. 1b, the theoretical PCE limits for multi-junction solar cells increase with the number of junctions, reaching 45.9% for double-junction structures, 51.8% for triple-junction structures, and up to 59.2% for six-junction structures [15]. Notably, the PCE gains diminish with an increasing number of junctions, highlighting the triple-junction structure as an optimal balance between achieving a high PCE limit and managing device complexity. It is important to note that multi-junction solar cells enable high PCEs across various architectures, including two-terminal (monolithic), four-terminal, and even multi-terminal designs, as illustrated in Fig. 1c [16, 17]. Among these, the monolithic architecture stands out due to its advantages, such as fewer electrodes and transparent conductive layers, which contribute to achieving high PCE with low-cost [18]. Furthermore, the monolithic design simplifies wiring and packaging complexities, ensuring seamless compatibility with standard encapsulation used in mainstream single-junction c-Si PV modules. Given these benefits, this review will primarily focus on monolithic triple-junction structure.

Before 2020, multi-junction solar cells have been most successfully realized in III-V compound semiconductors [19, 20]. Remarkable PCEs have been achieved with multi-junction III-V solar cells, including 32.9% for two-junction cells [21], 37.9% for three-junction cells [22], 38.8% for five-junction cells [23], and 39.5% for six-junction cells [24]. However, the high cost of III-V solar cells remains a major limitation to their widespread application. Obtaining high-quality III-V absorbers with a high degree of crystallographic perfection requires expensive and time-consuming epitaxial growth techniques [11]. As a result, III-V multi-junction solar cells have predominantly been used in specialized applications where their advantage in PCE justifies the high BOSs, such as in space exploration and concentrator systems [25, 26]. Perovskites have recently emerged as a promising class of photovoltaic materials, which can fulfill the demands of multi-junction technology, due to their exceptional optoelectronic properties, including tunable bandgaps, high optical absorption coefficients, long carrier diffusion lengths, and high defect tolerance [27]. Moreover, perovskite solar cells (PSCs) offer a cost advantage superior to III-V solar cells, as they can be fabricated using low-cost manufacturing processes [28]. In light of these advancements, perovskite–silicon double-junction solar cells (PSDJSCs) have become a central focus for both academic research and industrial innovation [29, 30]. As shown in Fig. 1d, the PCE of PSDJSCs has surged from 23.6% in 2016 to 34.85%, surpassing that of their double-junction III-V counterparts [31]. Similarly, the PCE of perovskite–perovskite double-junction solar cells has increased dramatically from 18.1% in 2016 to 30.1% [31]. These significant advancements underscore the substantial potential of perovskite-based multi-junction architecture.

In light of these, perovskite-based triple-junction solar cells represent an undoubtedly promising research direction for next-generation energy solutions. Since the first report of a perovskite-based triple-junction tandem solar cell in 2018 [32], various configurations, including perovskite/perovskite/silicon, perovskite/perovskite/organic, and all-perovskite structures, have achieved PCEs of 27.6% [33], 19.4% [34], and 28.4% [35], respectively. Notably, the all-perovskite triple-junction tandem solar cell recently developed by Hu et al. achieved a PCE of 28.4% [35], the highest reported among all-perovskite-based triple-junction configurations. Compared to similar devices reported the previous year [36], this reflects a significant net efficiency increase of 3.3%, highlighting the promising efficiency potential of all-perovskite triple-junction tandems. Among these configurations, the perovskite/perovskite/silicon structure remains particularly attractive due to its superior PCE potential and seamless integration with mainstream c-Si PV technologies. Although the efficiency of PSTJSCs achieved so far is still lower than PSDJSC in the last two years. Typically, more work reported PSDJSC with over 30% in recent years [37–42]. Encouragingly, the rapid advancements in PSDJSCs have spurred increased research interest in perovskite/perovskite/silicon triple-junction solar cells (PSTJSCs) in 2024. The PCE of the PSTJSCs has exhibited a remarkable upward trend, as highlighted by the red dashed line in Fig. 1d. It is foreseeable that, with continued and sustained research efforts, PSTJSCs also have the potential to achieve further breakthroughs in efficiency to over 30% and higher. Given this context, an analytical review that examines the development and discusses the current challenges facing PSTJSCs would be highly beneficial for the continued progress of this technology, with the aim of achieving high PCE and long-term stability.

This work presents a comprehensive analysis of advancements in PSTJSCs. Section 2 focuses on the fundamental principles, including power loss mechanism, bandgap tunability of perovskite materials, and theoretical approaches to PSTJSCs. Section 3 provides an overview of the progress achieved in the development of this technology. Section 4 highlights critical challenges, such as photocurrent limitation, open-circuit voltage (*V*_OC_) deficits, and stability issues. Additionally, we outline corresponding strategies to address these issues. Finally, we propose future directions aimed at further enhancing the PCE and stability of PSTJSCs. We believe this work will contribute significantly to advancing PSTJSCs to a new level of performance and maturity.

## Fundamentals

### Power Losses

The PV process in solar cells involves the absorption of sunlight with energy exceeding the semiconductor bandgap, while excess energy is dissipated as thermalization loss. Photons with energy below the bandgap are not absorbed and pass through the cell. Radiative recombination occurs due to photogenerated carriers, and at forward operating voltages, the carrier energy is defined by the quasi-Fermi level (QFL) separation, which determines the solar cell voltage at the electrical contacts [11].

**Fig. 2:**
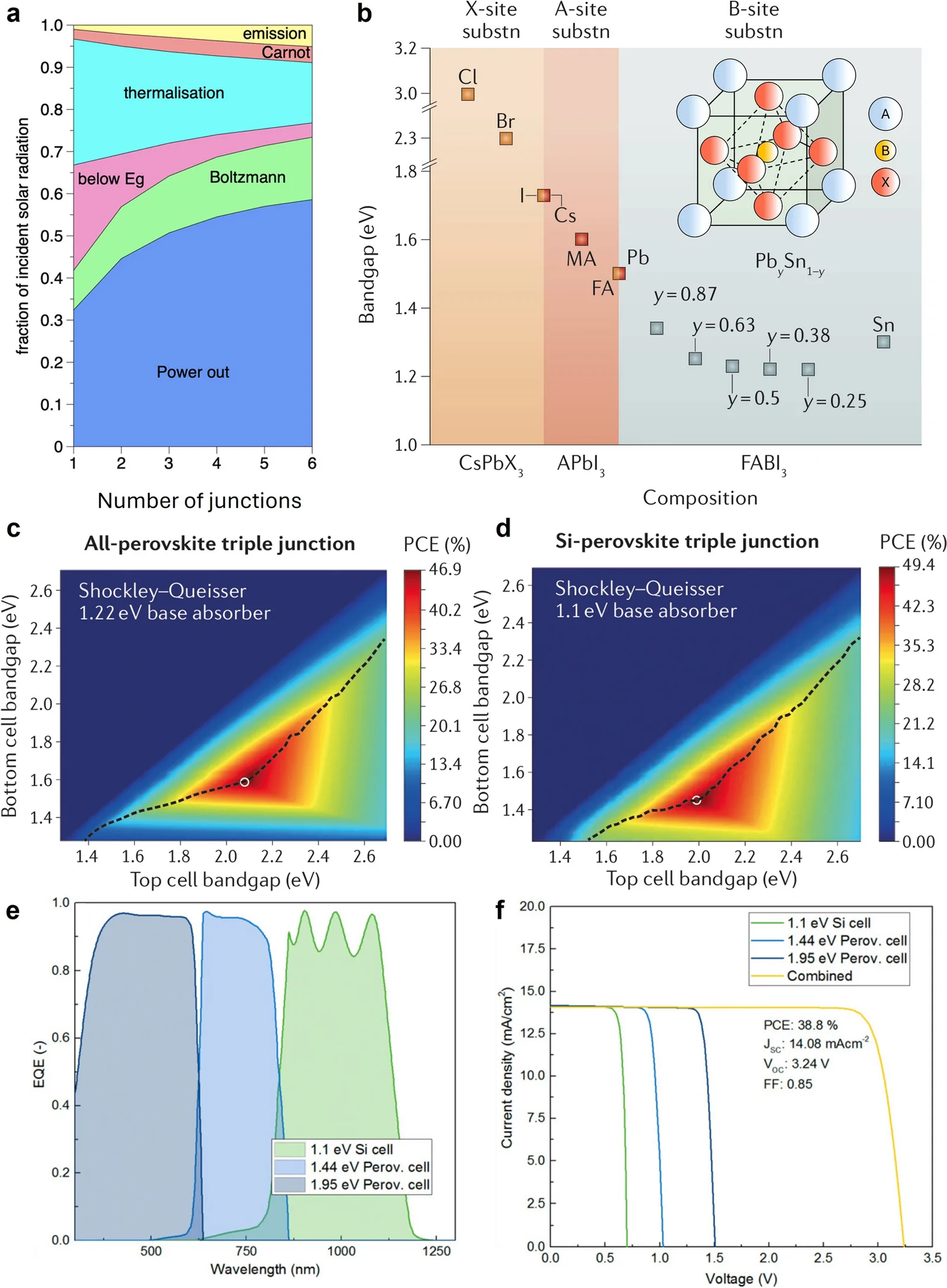
**a** Depiction of loss mechanisms and power output for an unconstrained multi-junction (MJ) solar cell under one-sun illumination (6000 K blackbody). Reproduced with permission from Ref. [43]. Copyright 2011 Wiley. **b** Graph showing the range of bandgaps achievable for tin and lead-based perovskite halides through lattice component substitution, with the inset illustrating the crystal structure of perovskite materials. Reproduced with permission from Ref. [44]. Copyright 2017 Springer Nature. PCE limits are calculated for triple-junction solar cells with **c** 1.22 eV and **d** 1.1 eV bottom absorbers. The dashed line represents the maximum PCE across all front-cell bandgaps, while the white circles identify the bandgap combinations yielding peak efficiency in each case. Calculated **e** external quantum efficiencies (EQEs) and **f** current–voltage curves of an optimized perovskite/perovskite/silicon solar cell with an ideal bandgap combination of 1.95, 1.44, and 1.1 eV. Reproduced with permission from Ref. [45]. Copyright 2017 American Chemical Society

Figure 2a outlines the distribution of power generation and losses in solar cells. In single-junction cells, the dominant losses are due to thermalization and unabsorbed sub-bandgap photons. Multi-junction solar cells reduce these losses by employing multiple semiconductors with different bandgaps, allowing for more efficient spectral absorption. However, other fundamental losses increase with additional junctions [43]. Boltzmann loss is an entropic loss linked to the re-emission of light, resulting in voltage loss due to increased optical mode occupancy. This loss can be reduced through solar concentration or by limiting radiative emission. In multi-junction cells, dividing the solar spectrum across multiple junctions leads to emission at various wavelengths, increasing Boltzmann loss. Carnot loss arises from the thermal excitation of carriers within bands and increases as additional junctions create more photogenerated carrier bands. This loss is generally unavoidable, except in rare cases where the solar cell operates at very low temperatures. Emission loss is intrinsic to solar cells due to the reciprocity between absorption and emission, as described by Kirchhoff’s law of radiation [46]. In optimized ideal multi-junction cells, emissive losses are minimal. However, in series-connected monolithic tandem configurations, where the current is mismatched, significant energy transfer may occur through radiative coupling.

Efficient operation in multi-junction solar cells requires closely matched photogeneration rates among subcells. This balance can be achieved by tuning the thickness and bandgap of absorbers, allowing overperforming subcells to transmit excess light to lower layers [47]. In radiatively efficient materials, excess photogeneration in subcells may radiate energy to lower junctions, with minimal escape from the top. It is worth mentioning that radiative coupling, observed in some perovskite materials, can mitigate spectral mismatches under blue-rich conditions and offers flexibility in bandgap configurations [48]. This flexibility enables the efficient operation of wide-bandgap perovskite top cells in silicon-based tandems [49].

### Bandgap Tunability of Perovskite

The ABX_3_ crystal structure of perovskite materials features an A-site cation (e.g., cesium (Cs⁺), methylammonium (MA⁺), or formamidinium (FA⁺)) within a cuboctahedral void, a B-site divalent metal (lead (Pb^2+^) or tin (Sn^2+^)) in a BX_6_ octahedron, and an X-site halide anion (chloride (Cl⁻), bromide (Br⁻), or iodide (I⁻)), superhalide, or pseudohalide (as shown in Fig. 2b**, inset**) [50]. A significant advantage of perovskites in multi-junction solar cells is their tunable bandgap, ranging from 1.2 to 3.0 eV, achieved by modifying the composition of the ‘A’ , ‘B’ and ‘X’ ions, as illustrated in Fig. 2b [44]. The bandgap of perovskites is primarily determined by the B- and X-site ions that form the [BX_6_] octahedral framework. The valence band maximum (VBM) arises from the coupling of B-site s orbitals and X-site p orbitals, while the conduction band minimum (CBM) is predominantly influenced by contributions from B-site p orbitals. The ‘A’ cations influence the bandgap by distorting the perovskite lattice, which alters the B-X bond length and angle [51–53]. Common ‘B’ cations, such as Sn^2+^, Pb^2+^, and germanium (Ge^2^⁺), exhibit bridging angles of 155.2°, 159.6°, and 166.3°, respectively, with larger angles corresponding to smaller bandgaps (APbX_3_ > ASnX_3_ > AGeX_3_) [54–57]. Similarly, increasing the electronegativity of the halogen ‘X’ anions (Cl⁻, Br⁻, I⁻) raises the bandgap (ABCl_3_ > ABBr_3_ > ABI_3_) [58]. Among these, halide tunability between Br and I offers a simple and effective method for adjusting the bandgap. For instance, methylammonium lead tri-halide (MAPbX_3_) can have its bandgap adjusted from 1.6 to 2.3 eV by altering the iodine-to-bromine ratio, while formamidinium lead tri-halide (FAPbX_3_) can be tuned from 1.48 to 2.23 eV using the same approach [59, 60].

However, a wider bandgap in perovskite materials does not necessarily translate to higher cell photovoltages. Wide-bandgap perovskites are susceptible to phase segregation, leading to I-rich domains that act as recombination traps and cause significant voltage deficits [59]. Conversely, tuning perovskites to bandgaps below 1.5 eV poses challenges, particularly when substituting Sn^2+^ for Pb^2+^, due to conflicts between spin–orbit coupling and steric effects [61–63]. Tin-based perovskite also introduces stability concerns, as Sn^2+^ is prone to oxidation to Sn^4+^, which undermines both stability and solar cell performance [57, 64]. These issues will be discussed in detail in a later section.

### Theoretical Approaches

In recent years, computational studies using optical models have been driven by the need to predict achievable PCEs before undertaking the complex fabrication of multi-junction devices. For monolithic devices, achieving current-matching conditions is crucial for maximizing energy conversion. Predicting optimized layer thicknesses can significantly enhance performance. The transfer matrix method (TMM) is particularly effective for modeling electromagnetic wave propagation through device layers, utilizing interface matrices with Fresnel coefficients and dissipation matrices incorporating Beer–Lambert absorption [65, 66].

Using the TMM, the theoretical PCE potential of perovskite-based triple-junction solar cells is assessed [44, 45]. As shown in Fig. 2c, d, detailed balance limits are plotted for varying bandgaps in the middle and top absorbers, focusing on two configurations: an all-perovskite triple-junction cell with a 1.22-eV perovskite as the rear absorber and a PSTJSC with a 1.1-eV silicon rear absorber. The all-perovskite triple junction demonstrates a theoretical PCE of 46.9% when absorber layers with bandgaps of ~ 2.1, ~ 1.6, and 1.22 eV are combined. This represents only a slight improvement over the SQ limit for all-perovskite double-junction solar cells (46.0%). In contrast, incorporating a silicon rear absorber with a lower bandgap enhances the PCE to 49.4% when combined with ~ 1.5 and ~ 2.0 eV bandgaps, delivering a significant PCE boost. Practical PCEs often fall below theoretical limits. Using TMM and device modeling, Hörantner et al*.* estimated more realistic PCEs [45]. Optimizing bandgap alignment and layer thicknesses yielded modest gains for all-perovskite triple-junction cells (32.2 to 33.0%), suggesting limited practicality. Conversely, as shown in Fig. 2e, f, an optimized PSTJSC achieved a PCE of 38.8% when absorber layers with bandgaps of 1.95, 1.44, and 1.12 eV, respectively. In addition to optical simulations, the authors conducted energy yield (EY) simulations under real-world climatic conditions representative of the Mohave Desert and Seattle, following a methodology similar to that reported by Hörantner and Snaith [67]. Their findings demonstrate that monolithically integrated tandem devices exhibit higher energy yields compared to both single-junction perovskite solar cells and previously modeled single-junction cells under the same environmental conditions. Notably, the tandem devices do not incur substantial additional losses from current-matching limitations, underscoring the potential performance advantage of PSTJSCs over other types of solar cells.

Furthermore, the modeling and simulation landscape for perovskite-based solar cells is progressively maturing, especially in the domains of numerical methodology, optical management, and energy yield prediction. A range of computational techniques have been widely adopted to solve the underlying electromagnetic problems, including the finite element method (FEM) [68, 69], finite-difference time-domain (FDTD) method [70, 71], and time-domain integral equation (TDIE) approach [72]. Integration of these complementary numerical techniques is increasingly recommended to enhance simulation accuracy and comprehensiveness for PSTJSC optimization.

## Roadmaps

Since the first device for PSTJSCs was reported in 2018, this field has attracted growing research interest. Particularly in 2024, significant advancements in PSDJSCs have catalyzed rapid progress in developing PSTJSCs. These progresses, which focus on optimizing current matching in the triple-junction and wide-bandgap perovskite top cells, have led to notable PCE milestones, positioning these devices as a remarkable step in solar energy technology.

**Fig. 3:**
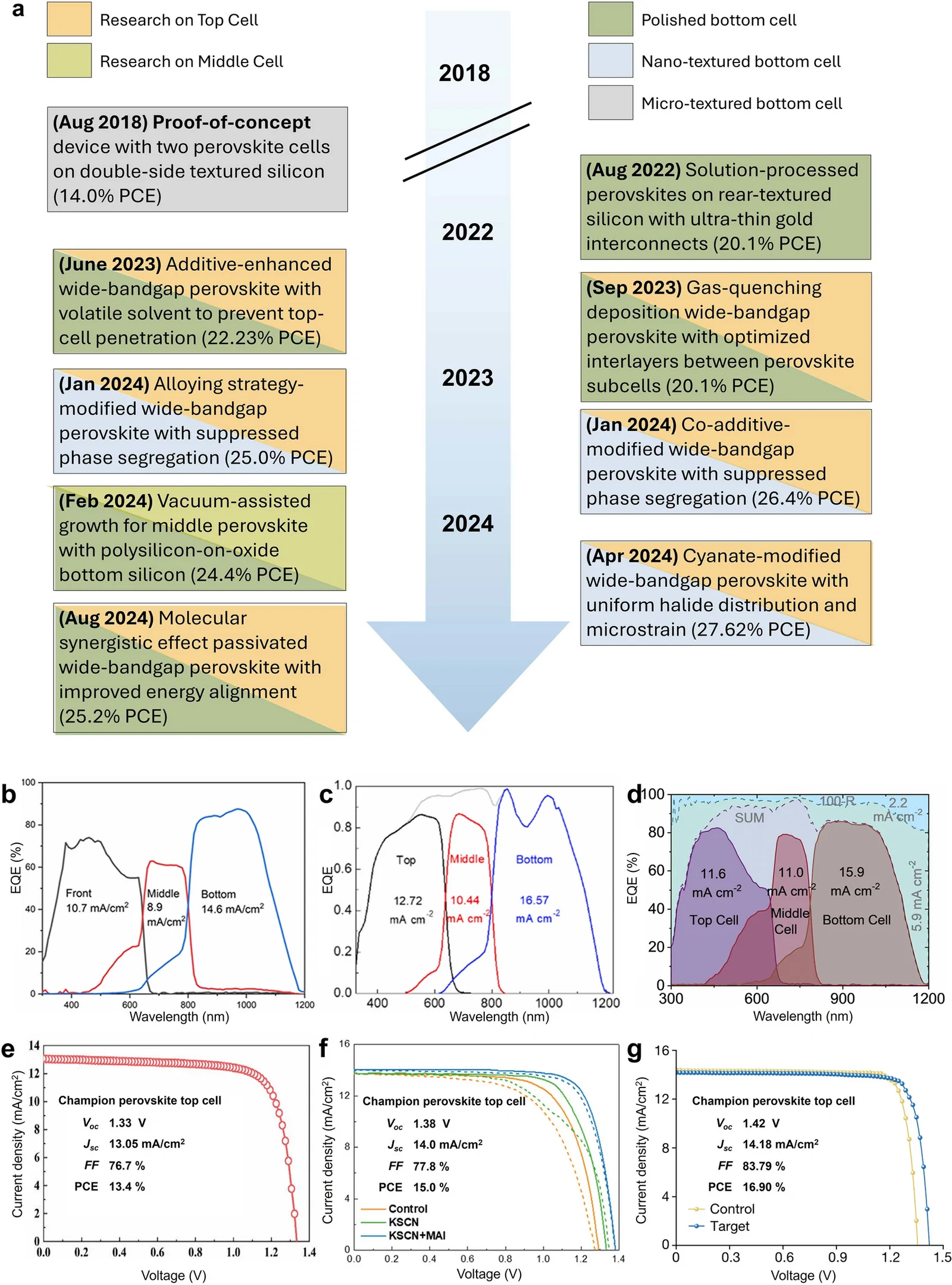
**a** Timeline highlighting the developments in perovskite/perovskite/silicon triple-junction solar cells, focusing on progress in top cells, middle cells, and interconnection layers. External quantum efficiency (EQE) curves for reported perovskite/perovskite/silicon triple-junction solar cells in **b** Zheng’s work, **c** Choi’s work, and **d** Hu’s work. Reproduced with permission from Ref. [73] Copyright 2022 American Chemical Society. Reproduced with permission from Ref. [74] Copyright 2023 American Chemical Society. Reproduced with permission from Ref. [75]. Copyright 2024 The Royal Society of Chemistry. Current–density to voltage (*J*-*V*) curves for reported champion wide-bandgap perovskite top cells in **e** Li’s work, **f** Xu’s work, and **g** Liu’s work. Reproduced with permission from Ref. [76]. Copyright 2024 John Wiley & Sons. Reproduced with permission from Ref. [77]. Copyright 2024 John Wiley & Sons. Reproduced with permission from Ref. [33]. Copyright 2024 Springer Nature

Figure 3a illustrates a timeline highlighting the progress in PSTJSCs, with their respective device structures and photovoltaic performance detailed in Table 1. In 2018, Werner et al*.* presented the first proof-of-concept device for PSTJSCs, achieving a PCE of 14% and an *V*_OC_ of 2.69 V [32]. This device utilized a double-side textured heterojunction (HJT) silicon bottom cell with micrometer-scale pyramidal structures to optimize light management. To ensure compatibility with the textured silicon substrate, both the mid-bandgap and wide-bandgap perovskite layers were fabricated using a hybrid vacuum and solution-based two-step deposition method. However, the device exhibited a low short-circuit current density (*J*_*SC*_) of 7.7 mA cm^−2^ and a low fill factor (*FF*) of 0.68. These are mainly attributed to the unoptimized current matching and the low quality of perovskite layers. While the hybrid two-step deposition method ensures the conformality of perovskite films on textured surfaces, it often results in relatively low film quality with small grains [78]. These defects are typically associated with reduced *V*_OC_ and *FF* in PSCs [79]. In 2022, Zheng et al*.* reported the second PSTJSC, achieving a PCE exceeding 20% and a *V*_*OC*_ of 2.74 V [73]. Unlike the earlier work, this study utilized a front-polished, rear-textured HJT silicon bottom cell. Both the mid-bandgap and wide-bandgap perovskite layers were deposited using a well-optimized one-step solution process. Additionally, an ultra-thin gold layer was introduced as part of the interconnection layer between the middle and top cells. These modifications significantly enhanced the *FF* of the device to an impressive value of 0.86. Nevertheless, the current matching in this work remained suboptimal, as indicated by the relatively low *J*_*sc*_ of 8.5 mA cm^−2^ limited by the middle-bandgap subcell (Fig. 3b).

**Table 1:** Summary of the device structure and photovoltaic performance for reported perovskite/perovskite/silicon triple-junction cells

Year	Top/Middle/Bottom Absorber	Top–Middle Interconnection layer	Middle–Bottom Interconnection layer	*V*_OC_ (V)	*J*_SC_ (mA/cm^2^)	FF	PCE (%)	Area (cm^2^)	Refs
2018	Cs_x_FA_1-x_Pb(I,Br)_3_ (1.80 eV)	IZO (150 nm)	nc-Si:H (p +)/nc-Si:H (n +)	2.69	7.7	0.68	14.00	1.4	[32]
	Cs_x_FA_1-x_Pb(I,Br)_3_ (1.53 eV)								
	Double-side micro-textured HJT Si cells								
2022	Cs_0.2_FA_0.8_Pb(I_0.45_Br_0.55_)_3_ (1.90 eV)	Au (1 nm)	ITO (20 nm)	2.74	8.5	0.86	20.10	1.0	[73]
	Cs_0.1_FA_0.9_PbI_3_ (1.55 eV)								
	Polished HJT Si cells								
2023	MAPb(I_0.5_Br_0.35_Cl_0.15_)_3_ (1.96 eV)	ITO (20 nm)	ITO (20 nm)	2.78	10.2	0.79	22.23	0.2	[74]
	Cs_0.1_FA_0.85_MA_0.05_PbI_3_ (1.56 eV)								
	n/a								
2023	Cs_0.05_(FA_0.55_MA_0.45_)_0.95_Pb(I_0.55_Br_0.45_)_3_ (1.83 eV)	ITO (15 nm)	ITO (20 nm)	2.87	8.9	0.78	20.10	1.0	[81]
	Cs_0.05_(FA_0.9_MA_0.1_)_0.95_Pb(I_0.95_Br_0.05_)_3_ (1.56 eV)								
	Polished textured HJT Si cells								
2024	Rb_0.05_Cs_0.12_FA_0.83_PbIBr_2_ (1.96 eV)	IZO (60 nm)	ITO (10 nm)	3.00	11.8	0.71	25.00 (24.19 certified, reverse scan)	1.0	[76]
	Cs_0.05_(FA_0.98_MA_0.02_)_0.95_Pb(I_0.98_Br_0.02_)_3_ (1.53 eV)								
	Double-side nano-textured HJT Si cells								
2024	Cs_0.1_FA_0.9_PbBr_2.1_I_0.9_ (2.0 eV)	IZO (35 nm)	IZO (20 nm)	3.04	11.9	0.73	26.40	1.0	[77]
	Rb_0.05_Cs_0.1_FA_0.85_PbI_3_ (1.52 eV)								
	Double-side nano-textured HJT Si cells								
2024	Cs_0.2_FA_0.8_Pb(I_0.5_Br_0.5_)_3_ (1.84 eV)	ITO (15 nm)	ITO (20 nm)	2.84	11.6	0.74	24.40	0.5	[75]
	FAPbI_3_ (1.52 eV)								
	Double-side polished POLO (polysiliconon– Passivating–oxide) Si cells								
2024	Cs_0.25_FA_0.6_MA_0.15_Pb(I_0.45_Br_0.5_OCN_0.05_)_3_ (1.93 eV)	ITO (8 nm)	ITO (15 nm)	3.13	11.6	0.76	27.62 (27.10 certified, steady state)	1.0	[33]
	Cs_0.1_FA_0.9_PbI_3_ (1.55 eV)								
	Double-side nano-textured HJT Si cells								
2024	Cs_0.05_(FA_0.85_MA_0.15_)_0.95_Pb(I_0.45_Br_0.55_)_3_ (1.95 eV)	IZO (60 nm)	ITO (20 nm)	3.07	11.4	0.72	25.17	1.0	[82]
	Cs_0.05_(FA_0.98_MA_0.02_)_0.95_Pb(I_0.98_Br_0.02_)_3_ (1.55 eV)								
	Rear-side textured tunnel oxide passivating contact (TOPCON) Si cells								
2025	Cs_0.05_(FA_0.55_MA_0.45_)_0.95_Pb(I_0.55_Br_0.45_)_3_ (1.83 eV)	ITO (5 nm)	ITO (80 nm)	3.00	9.0	0.80	21.50	1.0	[83]
	(FA_0.90_MA_0.10_)_0.95_Pb(I_0.95_Br_0.05_)_3_ (1.56 eV)								
	Rear-side textured HJT Si cells								
2025	MA_0.04_(Cs_0.2_FA_0.8_)_0.96_Pb(I_0.3_Br_0.66_Cl_0.04_)_3_ (1.83 eV)	IZO (~ 70 nm/60 Ω/square)	ITO (10 nm)	2.96	10.7	0.64	20.05	1.0	[84]
	Cs_0.1_FA_0.9_PbI_3_ (1.55 eV)								
	Double-side textured HJT Si cells								

Building upon Zheng’s device structure, Choi et al*.* made significant advancements in 2023 [74]. They found that one-step solution deposition of the wide-bandgap perovskite top layer could damage the underlying mid-bandgap perovskite cell, as the polar solvent might penetrate the interconnection layer and disturb the mid-bandgap perovskite film. To address this issue, they developed a novel solvent system comprising acetonitrile (ACN) and methylamine (MA) to deposit the wide-bandgap perovskite layer, successfully preventing such damage. Additionally, they increased the bandgap of the top perovskite layer to 1.96 eV, improving current matching within the device, as shown in Fig. 3c. As a result, their device achieved a PCE of 22.23%, a *V*_*OC*_ of 2.78 V, an *FF* of 0.79, and a *J*_*SC*_ exceeding 10 mA cm^−2^. In 2024, Hu et al. modified the mid-bandgap cell in PSTJSCs to achieve improved current matching [75]. They introduced a vacuum-growth method to enhance the quality of the medium-bandgap perovskite film, resulting in a stable, high-quality FAPbI₃ perovskite thin film, free of wrinkles, cracks, and pinholes, with a bandgap of 1.52 eV. As a result, their fabricated device demonstrated a PCE of 24.4%, with an improved *J*_*SC*_ of 11.6 mA cm^−2^ (Fig. 3d). In the meantime, Li et al*.* further improved the light management of their PSTJSCs by utilizing a double-side textured HJT silicon bottom cell with reduced pyramid sizes of less than 1 μm [76]. Unlike Werner‘s work, this type of bottom cell with reduced texture size is compatible with the one-step solution process, ensuring the deposition of high-quality perovskite films. Additionally, they improved the composition of the 1.96 eV wide-bandgap perovskite by alloying with Rb⁺ and Cl⁻ ions, which have small ionic radii. As a result, their device achieved a *V*_OC_ exceeding 3 V and a remarkable *J*_*SC*_ of 11.76 mA cm^−2^, leading to a PCE of 25%. In this study, the optimized wide-bandgap perovskite top cell demonstrates a PCE of 13.4%, a *V*_*OC*_ of 1.33 V, and an *FF* of 0.77, as shown in Fig. 3e, which remains relatively low in performance. According to the SQ limit, the maximum *V*_OC_ for a 1.96 eV perovskite is theoretically up to 1.6 V, indicating a significant *V*_OC_ deficit of 0.63 eV in their current device [8, 80].

**Fig. 4:**
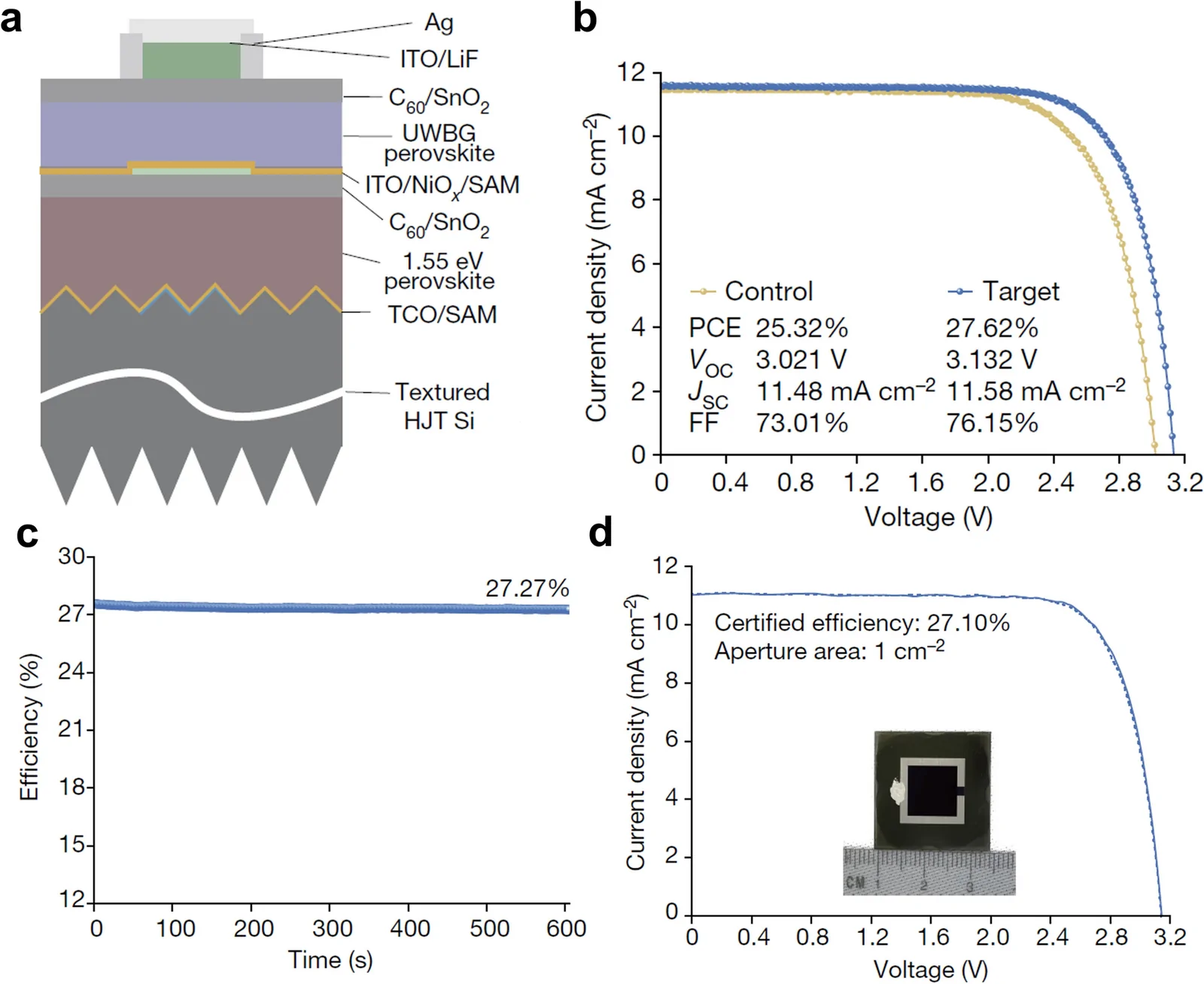
**a** Cell structure, **b** in-house measured *J*-*V* curve, **c** MPP tracking measurement and **d** certified *J–V* curves of record monolithic PSTJSCs. Reproduced with permission from Ref. [33]. Copyright 2024 Springer Nature

Recently, Xu et al*.* proposed modifying the wide-bandgap perovskite by incorporating potassium thiocyanate (KSCN) and methylammonium iodide (MAI) co-additives to improve the film quality and reduce the *V*_OC_ deficit [77]. They demonstrated that SCN^−^ enhances the perovskite grain size, thereby reducing the grain boundary defect density; K⁺ stabilizes the halide, preventing halide vacancy formation; and MA⁺ removes residual light-destabilizing SCN^−^ from the perovskite films through double displacement reactions. As a result, their wide-bandgap perovskite top cell achieved a significantly enhanced *V*_OC_ of 1.38 V and a PCE of 15.0%, as shown in Fig. 3f. These improvements contributed to a *V*_OC_ of 3.04 V and a PCE of 26.4% in their PSTJSC. More recently, Liu et al. introduced cyanate (OCN⁻), a previously unexplored pseudohalide, as an alternative to bromide in perovskite compositions [33]. With an effective ionic radius of 1.97 Å, closely matching that of bromide (1.95 Å), OCN⁻ incorporation into the perovskite lattice was shown to create a uniform halide distribution and minimize microstrain. These improvements significantly enhanced the quality of wide-bandgap perovskite films. As a result, their 1.93 eV wide-bandgap perovskite top cell achieved a notable *V*_OC_ of 1.42 V, as shown in Fig. 3g. Consequently, their PSTJSC demonstrated a *V*_OC_ surpassing 3.1 V and achieved a record PCE of 27.62% (certified 27.10% on 1 cm^2^) as shown in Fig. 4.

It is worth noting that the PSTJSC involves two different interconnection layers between the subcells: a top–middle subcell (perovskite–perovskite) interlayer and a middle–bottom subcell (perovskite–Si) interlayer. Table 1 also summarizes the interconnection layers used in reported PSTJSCs. For the middle–bottom subcell interlayer, with the exception of the first demonstration of PSTJSCs that employed a stacked nc-Si:H (p⁺)/nc-Si:H (n⁺) structure [32], all subsequent demonstrations have utilized a transparent conductive oxide (TCO) layer, typically either indium tin oxide (ITO) [33, 73–76, 81–84] or indium zinc oxide (IZO) [77]. This approach follows a similar optimization trend as that observed in PSDJSCs, with commonly adopted TCO thicknesses ranging from 10 to 20 nm for high performance PSDJSCs [12, 37, 85–89]. For the top–middle interlayer (perovskite–perovskite), Zheng et al. employed an ultra-thin 1 nm gold (Au) layer as the interconnection layer [73]. Other demonstrations used a TCO interlayer, either ITO or IZO. The use of the ultra-thin Au interlayer led to a high FF but a lower *J*_SC_ compared to TCO-based tandem solar cells [73]. This reduction in *J*_SC_ may be also attributed to the parasitic absorption introduced by the thin metal layer. However, there is potential for further improvement if the metal thickness can be further optimized, as reported in studies on high performance perovskite–perovskite double-junction solar cells [90]. Among the TCO-based interlayers for the top–middle subcell, the record PSTJSC employed an 8 nm thin ITO layer [33]. Compared with other PSTJSCs, this demonstration achieved both higher FF and *J*_SC_, indicating a promising strategy. Future interlayer optimizations may devote to further optimize the thickness of TCO layers [91] or exploring ultra-thin metal layers [90], typically for top–middle interlayer, to achieve enhanced optical and electrical performance for better performance of PSTJSCs.

In summary, since the initial report of the proof-of-concept device, research efforts from 2018 to 2023 primarily contributed to efficiency improvements by optimizing the photocurrent for higher *J*_SC_ in triple-junction configurations. These efforts included tuning the bandgap of the perovskite top cell, enhancing the film quality of the perovskite middle cell, and modifying the surface structure of the silicon bottom cell. As a result, the *J*_SC_ of PSTJSCs increased significantly, from an initial 7.7 to over 11.5 mA cm^−2^. However, a* J*_*SC*_ of 12 mA cm^−2^ appears to represent an upper limit for the current device architecture. All studies reported in 2024 show *J*_*SC*_ values below this threshold, largely constrained by the current-limiting medium-bandgap perovskite middle cell, as indicated by the EQE curves shown in Fig. 3b-d. To further enhance the *J*_*SC*_ of PSTJSCs, strategies such as further lowering the bandgap of the medium-bandgap perovskite need to be explored, which will be discussed in detail in the following sections. In 2024, research primarily focused on optimizing the wide-bandgap perovskite top cell to reduce the* V*_*OC*_ deficit. Notably, Liu’s work achieved a* V*_*OC*_ deficit of 0.51 eV for wide-bandgap perovskite solar cells, which is the lowest reported for bandgaps exceeding 1.9 eV [33]. However, compared to medium-bandgap perovskite solar cells, with bandgaps between 1.5 and 1.6 eV, which typically exhibit a *V*_*OC*_ deficit below 0.4 eV, there remains significant room for improvement [92]. These challenges and their corresponding strategies will be detailed in the later section.

## Challenges and Strategies

### Current Mismatch

As discussed in Sect. 2, a well-optimized PSTJSC has the potential to achieve a *J*_SC_ exceeding 14 mA cm^−2^. However, reported studies to date rarely demonstrate *J*_SC_ values surpassing 11.5 mA cm^−2^, highlighting a significant gap. This photocurrent limitation poses a major obstacle, severely restricting the ability of PSTJSCs to achieve their full performance potential. A significant challenge for PSTJSCs lies in addressing the issue of the current mismatch. In an ideal PSTJSC, the perovskite middle subcell paired with a 1.1-eV silicon bottom cell should have a bandgap of approximately 1.44 eV [45]. However, reported triple-junction devices typically feature middle subcells with a minimum bandgap of 1.52 eV [77]. This discrepancy causes current mismatch, as the middle cell limits the overall current output of the device, while the silicon bottom cell generates the highest current, as illustrated in Fig. 3b-d. To overcome this limitation, further research is needed to reduce the bandgap of the middle cell, thereby increasing current density and enhancing the performance of PSTJSCs.

The use of FAPbI_3_-based perovskites with an ideal bandgap of approximately 1.47–1.53 eV presents a promising option for mid absorbers in PSTJSCs [93]. To date, single-junction FAPbI_3_ PSCs have demonstrated exceptional performance, achieving PCEs of 25.8 and 24.1% in n-i-p and p-i-n device architectures, respectively [94, 95]. However, the primary challenge associated with FAPbI_3_ lies in its limited structural stability [96]. The disordered interaction between FA^+^ and I^−^ ions causes the asymmetrical FA^+^ cation to adopt an off-centered position, leading to the formation of a trigonal structure instead of the desired cubic phase [97]. Experimentally, low-temperature annealing around 100 °C often results in the formation of a one-dimensional (1D) yellow non-perovskite polymorph (yellow δ-phase). Additionally, exposure to high humidity can trigger a phase transition from the black α-phase to the yellow δ-phase. This structural distortion can alter the bandgap of the FAPbI_3_ film and reduce photocurrent generation [98, 99]. For instance, in Hu’s study, the reported FAPbI_3_ film exhibited a bandgap of 1.52 eV, higher than the ideal 1.47 eV, thus perpetuating current mismatch issues in PSTJSCs [77]. Addressing these challenges will require ongoing research efforts, including compositional engineering, defect passivation, and process optimization [100–103]. Comprehensive insights into FAPbI_3_-based perovskites and strategies to overcome these limitations are listed in recent review articles [104–106].

Further lowering the bandgap of perovskites can be achieved through the complete or partial substitution of Pb^2+^ with Sn^2+^ in the B-site cation, which directly influences the conduction band [107, 108]. Sn-containing perovskites with mixed halides (Br/I) exhibit a broad bandgap range from 1.2 to 2.0 eV [109]. However, these absorbers are predominantly utilized as low-bandgap materials for the bottom cells in all-perovskite tandem devices [110, 111]. Consequently, research has primarily focused on Sn-based perovskites with bandgaps around 1.2 eV, while limited studies address Sn-based perovskites with bandgaps above 1.35 eV, which are suitable for middle cells in triple-junction devices. Recently, Yang et al. developed a MAPb_0.5_Sn_0.5_(I_0.8_Br_0.2_)_3_ perovskite film with a bandgap of 1.35 eV [112]. By incorporating bromine and increasing the Sn ratio to 50%, they enhanced the optoelectronic properties of the perovskite absorber. These improvements reduced non-radiative recombination and increased the absorption coefficient, enabling their device to achieve a PCE of 17.63%. In addition to mixed Pb–Sn perovskites, pure Sn perovskites, such as FASnI_3_, present a compelling alternative [113]. With a bandgap of 1.40 eV, FASnI_3_ is well suited for the middle cell in PSTJSCs. Moreover, employing Pb-free perovskites mitigates concerns about Pb toxicity. Despite this potential, the highest certified PCE for FASnI_3_ solar cells remains 14.1%, lagging behind pure Pb and Pb–Sn perovskites [114]. The major challenge for Sn-based perovskites lies in their critical stability issues, primarily driven by oxidation of Sn^2+^ by atmospheric oxygen, a process exacerbated by moisture [115–117]. Additionally, the rapid crystallization of Sn perovskites, compared to their Pb counterparts, often results in poor film quality with low crystallinity [118]. To address these challenges, numerous studies have focused on enhancing both the PCE and stability of Sn-based perovskite solar cells, as detailed in recent reviews [119–122]. The application of Sn-based perovskites as middle cells in PSTJSCs holds promise for future advancements.

### Light Management

**Fig. 5:**
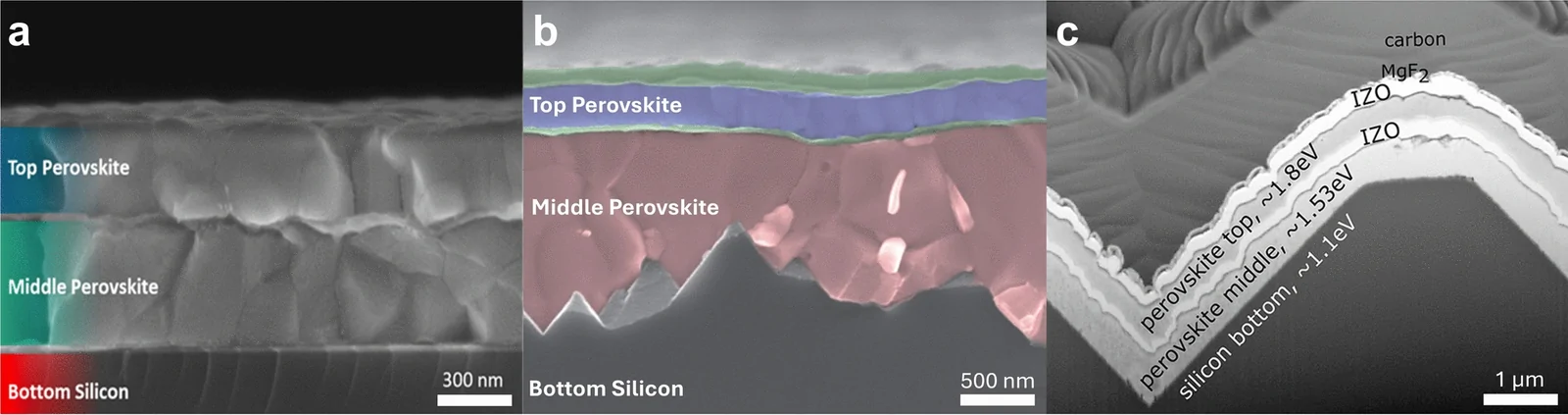
Cross-section scanning electron microscopy (SEM) images of PSTJSCs based on **a** polished, **b** nanotextured, and **c** micro-textured silicon bottom cells, respectively. Reproduced with permission from Ref. [81]. Copyright 2023 American Chemical Society. Reproduced with permission from Ref. [33]. Copyright 2024 Springer Nature. Reproduced with permission from Ref. [32]. Copyright 2018 American Chemical Society

In addition to improving current matching, enhancing light management within the device is another critical strategy, with the optics of the silicon bottom cell playing a pivotal role [123]. A polished silicon surface exposed to air typically exhibits a reflectance of 30%–40% [124]. This high reflectance leads to significant light loss, reducing the contribution to photocurrent generation. Historically, most PSDJSCs fabricated before 2018 utilized silicon bottom cells with polished front surfaces [125]. The cross-section scanning electron microscopy (SEM) image of some reported PSTJSCs based on polished silicon bottom cells has been shown as an example in Fig. 5a. While such surfaces offer the advantage of enabling conformal deposition of perovskite and carrier-selective layers, they suffer from three major limitations: (i) substantially higher reflection from the front silicon surface, (ii) diminished light trapping at longer wavelengths, and (iii) increased fabrication costs due to the additional polishing step [123].

To address this issue, texturing—a chemical etching process that modifies the surface’s physical structure—is commonly employed in silicon single-junction solar cells [126]. A typical industrially textured silicon surface has pyramid base size typically ranges from 3 to 8 μm. This surface, fully covered with pyramids, redirects reflected light predominantly back onto the silicon surface, allowing for a second absorption opportunity. As a result, the textured surface of a bare silicon wafer in air achieves significantly improved absorption, reducing reflection to approximately 10% [127]. While micron-sized pyramids are highly effective in minimizing reflection for single-junction silicon solar cells, they are less compatible with the solution-processing techniques used for perovskite top cells in PSTJSCs [128]. A viable alternative is to reduce the size of the surface pyramids, enhancing compatibility with solution-processed perovskite films [129, 130]. Although industry-standard micron-sized pyramids pose challenges for perovskite deposition, smaller pyramids enable conformal solution processing, as shown in Fig. 5b. As highlighted in Fig. 3a, two recent studies reporting PCEs exceeding 26% employed textured silicon bottom cells with reduced pyramid sizes. However, it is important to note the inherent trade-off: smaller pyramids improve compatibility with perovskite top cell processing but also result in increased reflection compared to standard micron-sized pyramids [131].

Another important strategy involves modifying the perovskite deposition technique. Instead of relying on solution-based processes, the perovskite layer can be deposited using evaporation or a hybrid evaporation solution two-step process, which is compatible with micro-textured silicon surfaces to maximize light trapping, as shown in Fig. 5c [132, 133]. However, these deposition methods are still relatively underdeveloped. For PSDJSCs, the PCE of devices employing the hybrid two-step or evaporation deposition process on micro-textured silicon bottom cells remains lower than that of devices fabricated using the solution-based method on polished or nano-textured silicon bottom cells [134, 135]. To date, only the initial proof-of-concept device has been fabricated using an industrially textured silicon surface with micron-sized pyramids, but it suffers from poor film quality [32]. Encouragingly, a growing number of studies have focused on advancing these methods, as highlighted in recent review articles [136–138]. Moving forward, increased research efforts are essential to further develop PSTJSCs that leverage micro-textured silicon surfaces.

### Voltage Deficit

**Fig. 6:**
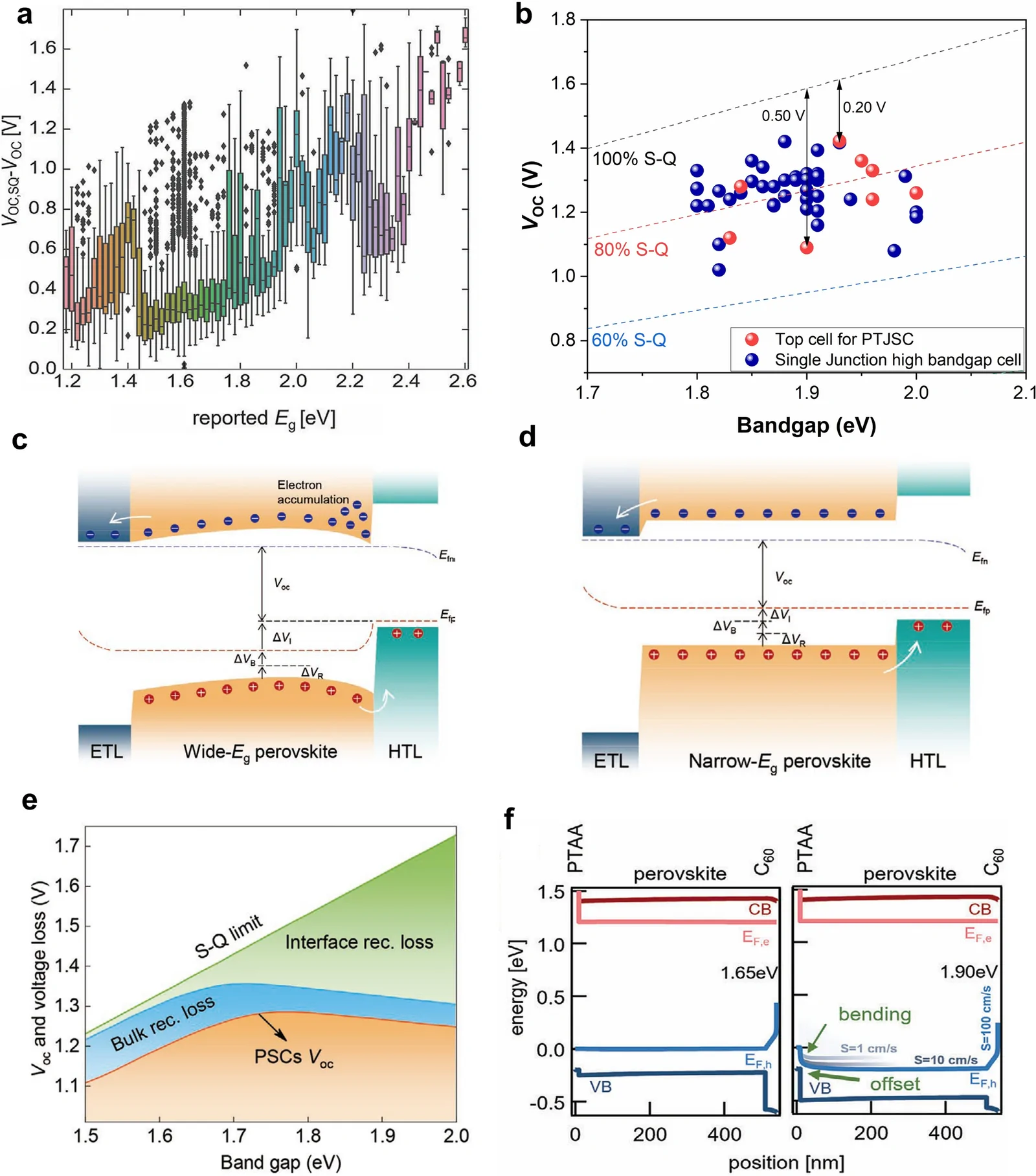
**a** Relationship between the *V*_OC_ loss, defined as *V*_OC,SQ_ − *V*_OC_, and the bandgap energy is illustrated for all cells included in the perovskite database. The graph also features the SQ limit (solid line) and the SQ limit reduced by 5% (dashed line) for comparison. Each data point is color coded to represent the publication date of the corresponding study. Reproduced with permission from Ref. [140]. Copyright 2024 John Wiley & Sons. **b** Reported photovoltaic parameters of perovskite solar cells with bandgaps from 1.8 to 2.0 eV [33, 73–77, 81, 82, 142], with dashed lines indicating fractions of the Shockley–Queisser (SQ) limits. **c**, **d** Energy level schematics for narrow- and wide-bandgap perovskites, illustrating radiative (ΔV_R_​), bulk (ΔV_B_​), and interface (ΔV_I_​) recombination losses. Reproduced with permission from Ref. [143]. Copyright 2022 John Wiley & Sons. **e** Voltage loss distribution and *V*_OC_​ of PSCs, highlighting interface (perovskite/ETL, perovskite/HTL) and bulk recombination losses. Reproduced with permission from Ref. [143]. **f** The band diagrams of PSCs with bandgaps of 1.65 eV (left) and 1.90 eV (right), employing fixed transport materials (undoped PTAA and C_60_). Reproduced with permission from Ref. [140]. Copyright 2024 John Wiley & Sons

In conventional solar cells, the theoretical maximum *V*_OC_ is expected to increase with a widening bandgap of the absorber. However, for wide-bandgap perovskites, especially those with bandgaps exceeding 1.7 eV, a photovoltage plateau is frequently observed in their solar cells [139]. Indeed, as discussed in Sect. 3, all reported wide-bandgap perovskite top cells for PSTJSCs are suffer from a large *V*_OC_ deficit. As illustrated in Fig. 6a, the highest-performing PSC for medium-bandgap perovskites (1.5–1.6 eV) exhibit relatively low *V*_OC_ deficits, typically around 0.3–0.4 V. In contrast, wide-bandgap PSCs (1.8–2.0 eV) often experience significantly higher *V*_OC_ deficits, exceeding 0.5 V [140]. In the case of the wide-bandgap top cell in PSTJSC applications, although the *V*_OC_ deficit has been reduced from the initial 0.5 V to the most recent 0.2 V, as shown in Fig. 6b, it remains a significant challenge for such bandgap perovskite devices. This substantial *V*_OC_ loss in wide-bandgap PSCs arises from several factors beyond the unavoidable SQ limit [139, 141]. These include: (i) suboptimal wide-bandgap perovskite film quality characterized by an increased density of defects, and (ii) mismatched energy levels and improper interfacial energetics at the perovskite and charge transport layer (CTL) interface. Addressing these challenges is critical for improving the *V*_OC_ of wide-bandgap perovskite top cells and the overall PCE of PSTJSCs.

Film quality of the perovskite encompasses various factors, including crystallinity, morphology, and phase stability. Poor film quality often results in a high density of charged defects in perovskites, particularly at the surface and grain boundaries, which can trap carriers. While some trapped charges may eventually escape and be collected by the electrodes—minimizing their impact on photocurrent output—the associated energy disorder and reduced carrier concentration can significantly lower the QFL splitting, thereby adversely affecting the *V*_OC_ [144]. For wide-bandgap perovskites suitable for PSTJSCs (1.8–2.0 eV), the Br content in the film is typically over 40%, significantly higher than that of medium-bandgap perovskites. The intrinsic differences between Br⁻ and I⁻—including variations in atomic radius, diffusivity, and binding interactions with solvents—can influence precursor formulation and add complexity to the crystallization process of thin films [145]. During the deposition and annealing stages of wide-bandgap perovskite film, the rapid crystallization rate of Br-rich perovskites often leads to non-uniform vertical halide distribution and a high density of defects [146].

While significant progress has been made in defect passivation strategies for PSCs with bandgaps between 1.5 and 1.6 eV, these approaches appear less effective for wide-bandgap PSCs, suggesting that the mechanisms behind energy losses may differ fundamentally. Since similar A-site and B-site cations are employed in these perovskites, the variation in defect characteristics is likely attributed to differences in the I:Br ratios. Previous studies on defects in perovskites have revealed that negative iodine interstitials (I_i_⁻) are unstable and prone to oxidation, forming their positive counterparts (I_i_⁺). This oxidation process results in comparable densities of I_i_⁺ and I_i_⁻, both of which can trap photogenerated electrons and holes through (+ /0) and (0/ −) transitions, respectively [147]. Furthermore, the increased bromine content in wide-bandgap perovskites leads to an expanded Br-Br (or Br-I) distance within the lattice. This occurs because the smaller ionic radius of Br increases the Pb-Br-Pb angle compared to Pb-I-Pb [148]. Consequently, the formation of I_i_⁺ defects is facilitated in Br-Br or Br-I regions. Although I_i_⁺ defects are relatively benign—since electron trapping by I_i_⁺ requires a substantial charge recombination barrier (~ 0.3 eV)—experimental findings indicate that these defects significantly limit the photovoltage of wide-bandgap PSCs [149]. So far, researchers have investigated various approaches, including additive engineering, solvent optimization, and surface passivation techniques, to improve the film quality of wide-bandgap perovskites. These strategies have been comprehensively discussed in recent review articles, which are expected to be applied in PSTJSCs in future [150–153].

On the other hand, compared to the extensively studied PSCs with medium bandgaps of 1.5–1.6 eV, wide-bandgap perovskites, which are ideal for PSTJSCs with bandgaps ranging from 1.9 to 2.0 eV, exhibit notable shifts in their electronic structure [152]. Specifically, the CBM shifts upward, while the VBM shifts downward as shown in Fig. 6c, d. These shifts lead to adjustments in the QFL when the wide-bandgap perovskite interfaces with CTLs to reach equilibrium [154]. The linear increase in *V*_OC_ loss observed with increasing bandgap (Fig. 6e) can thus be attributed to the increasing mismatch between the absorber material and the transport layers. Suchan et al*.* visualized the band diagrams of the simulated devices at open-circuit conditions, as shown in Fig. 6f [140]. These include a nearly perfectly aligned case (1.65 eV) and a highly misaligned case (1.90 eV). In the misaligned scenario, a noticeable offset exists between the valence band of the perovskite and the highest occupied molecular orbital (HOMO) level of the hole transport layer poly[bis(4-phenyl)(2,4,6-trimethylphenyl)amine (PTAA). This offset leads to significant electron accumulation and interfacial recombination, evident from the pronounced bending of the QFL for holes, which constrains the achievable *V*_OC_. Notably, the bulk material permits a much higher *V*_OC_, with approximately a 0.15-eV difference between the QFL in the bulk and at the contacts [140]. These findings underscore the critical importance of achieving optimal energy level alignment between the wide-bandgap perovskite and CTLs to maximize *V*_OC_. To date, some research efforts have been made to modify the energy levels of existing materials to address the mismatch issue [155, 156]. However, there is an urgent need for the development of novel charge transport materials specifically designed to mitigate energy level offsets effectively. In a word, minimizing *V*_OC_ loss in suitable bandgaps for PSTJSCs requires more focus on interfacial passivation and band alignmangent [143].

### Stability

Compared to well-established c-Si solar cells, which can endure rigorous standard testing conditions and demonstrate field durability exceeding 25 years when properly encapsulated, perovskite-based PV technology faces significant challenges in achieving comparable long-term stability [157]. The optoelectronic quality of perovskite absorbers often deteriorates irreversibly, depending on external degradation factors such as electrical bias, temperature, and environmental conditions [158, 159]. Fortunately, significant efforts have been directed toward addressing stability challenges within the perovskite research community in past few years [160–162]. For single-junction PSCs with medium bandgaps between 1.5 and 1.6 eV, substantial progress has been made, with the most advanced devices demonstrating remarkable stability improvements, sustaining performance over extended periods [162–165]. However, the studies on stability of wide-bandgap PSCs is lagging behind. Herein, within the scope, we mainly discussed the specific stability issues raised in PSTJSCs, especially the light instability in wide-bandgap PSCs.

**Fig. 7:**
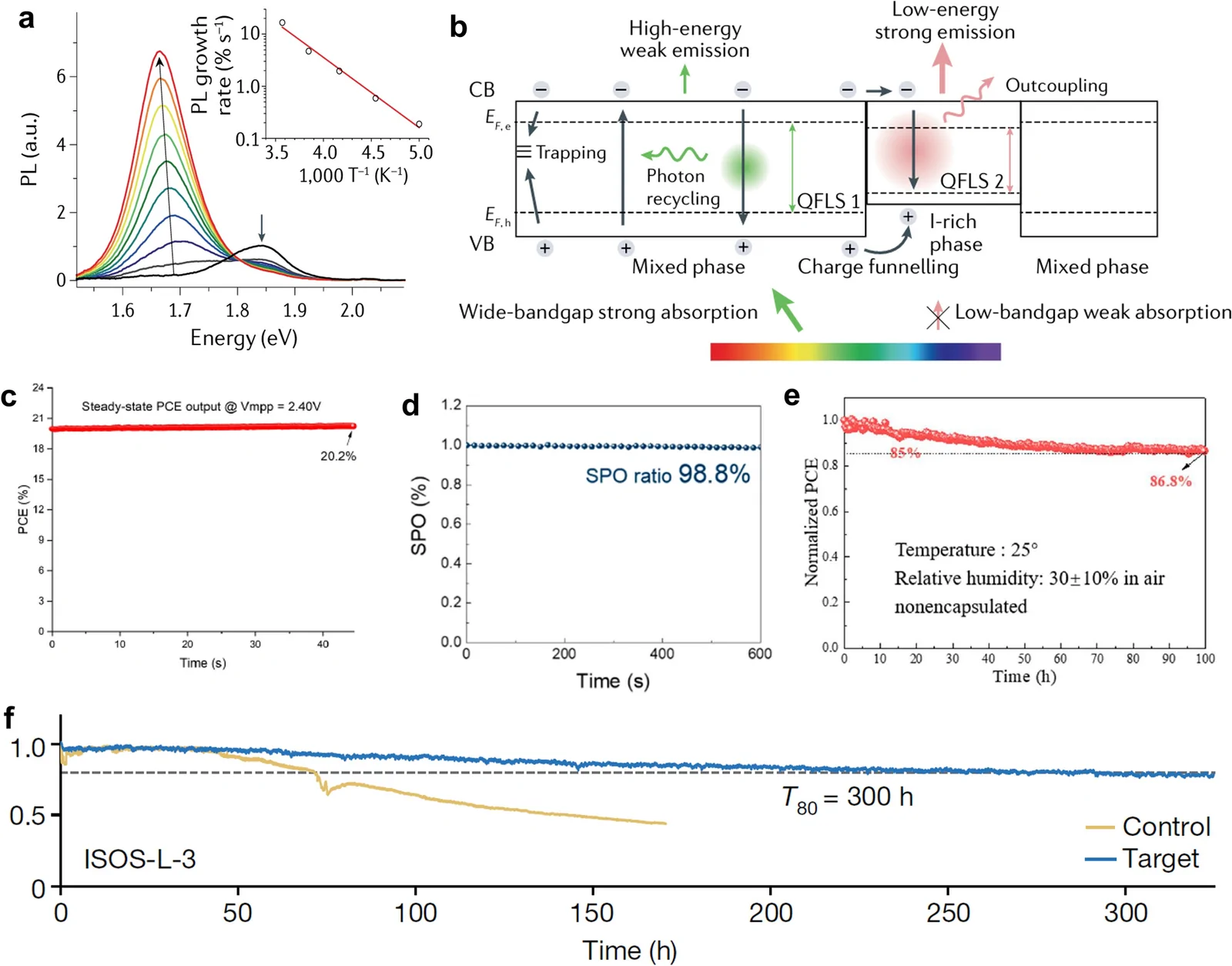
**a** Steady-state photoluminescence (PL) spectra of a wide-bandgap perovskite film with the composition MAPb(Br_0.4_I_0.6_)_3_, recorded over a duration of 45 s in 5-s increments under 457 nm illumination at 300 K. The inset depicts the temperature-dependent behavior of the initial PL growth rate around 1.68 eV. Reproduced with permission from Ref. [59]. Copyright 2015 The Royal Society of Chemistry. **b** Absorption, emission, and recombination processes in a halide-segregated perovskite system. Notations include a.u. (arbitrary unit), CB (conduction band), E_F,e_ (quasi-Fermi level for electrons), E_F,h_ (quasi-Fermi level for holes), and VB (valence band). Reproduced with permission from Ref. [166]. Copyright 2021 American Chemical Society. Stability track results under 1-sun illumination of reported PSTJSCs, **c** stead-state PCE under fixed voltage for 45 s. Reproduced with permission from Ref. [73]. Copyright 2022 American Chemical Society. **d** MPP tracking under continuous 1-sun illumination for 600 seconds. Reproduced with permission from [74] Copyright 2023 American Chemical Society. **e** MPP tracking results under 1 sun for 100 hours. Reproduced with permission from Ref. [76] Copyright 2024 John Wiley & Sons. **f** The best MPP tracking results are under 1 sun for 300 hours. Reproduced with permission from [33] Copyright 2024 Springer Nature

Mixed halide perovskites, particularly wide-bandgap variants with high Br content, often exhibit instability due to the disruption of homogeneously distributed halides under equilibrium conditions, as shown in Fig. 7a. In 2015, McGehee et al*.* introduced the concept of “phase segregation” in MAPb(I_*x*_Br_1-x_)_3_ perovskites for the first time, where light exposure triggers halide migration, resulting in I-rich and Br-rich regions within the material [59]. Over the past decade, several models have been proposed to explain halide segregation, including thermodynamic instability, chromatographic effects, polaron-induced lattice strain, charge trapping-induced electric fields, and stability discrepancies between perovskite phases [167]. However, none of them fully explained this phenomenon. This instability has been linked to significant photovoltage losses in wide-bandgap PSCs. It is suggested that charge carriers funneling from Br-rich to I-rich domains restricts the QFLS [168]. Given that the *V*_OC_ is typically extracted from *J-V* measurements conducted within seconds, it remains unclear whether photo-induced phase segregation occurs rapidly enough to substantially impact the device *V*_OC_ during such short timescales [169]. Nevertheless, the phenomenon of phase segregation can obviously cause the instability issue for the device operating under illumination. It creates sub-bandgap trap states, which elevate non-radiative recombination rates and disrupt the energy levels of the perovskite, leading to negative impacts on charge transport across the interfaces with CTLs, as illustrated in Fig. 7b [170]. Furthermore, changes in the optical bandgap can result in a mismatch between the photocurrent of the wide-bandgap perovskite top cell and other subcells, further compromising the overall stability and performance of the device [171].

Table 2 presents the stability data for all reported PSTJSCs. In 2022, Zheng et al. reported 45 s of MPP tracking under continuous 1-sun illumination in their fabricated PSTJSCs (Fig. 7c) [73]. After that, the reported PSTJSCs have undergone light stability tests of MPP tracking under continuous 1-sun illumination ranging from a few hundred seconds (Fig. 7d) to a hundred hours (Fig. 7e), during which the devices typically degrade rapidly. Recently, Liu et al. identified phase segregation in the wide-bandgap perovskite top cell as a key factor contributing to such poor light stability [33]. Their control devices, exhibiting strong phase segregation under illumination, degraded significantly—losing over 30% of its initial PCE within 300 h of MPP tracking under illumination (Fig. 7f). In contrast, their target device, designed to suppress phase segregation, demonstrated remarkable light stability, with no PCE loss after 300 h of the test. This finding highlights a substantial enhancement in the light stability of their fabricated PSTJSCs and underscores the critical role of phase segregation in determining the light stability of PSTJSCs. Herein, for achieving long-term operational stability, further investigation is needed to comprehensively understand and address the challenges posed by halide segregation in wide-bandgap perovskites. Encouragingly, beyond Liu’s work, several strategies have been developed to mitigate phase segregation. Incorporating an oxide-based buffer layer—such as ytterbium oxide (YbO_*x*_) can be a promising strategy [172]. These buffer layers can serve multiple functions, including facilitating efficient charge extraction, minimizing interfacial recombination losses, and acting as barriers to ion migration and environmental stressors [173]. In particular, YbO_*x*_ has demonstrated favorable energy level alignment, high transparency, and excellent thermal and chemical stability, making it well suited for integration into tandem architectures [172]. Its incorporation not only enhances device performance but also contributes to prolonging the operational lifetime of the solar cells, thereby addressing key challenges in the commercialization of perovskite devices. More strategies include compositional and stoichiometric engineering, morphological optimization, crystallinity control, and trap state passivation, as discussed in various comprehensive reviews [174–176]. These strategies effectively to PSTJSCs will be crucial for enhancing their operational stability for future research.

**Table 2:** Summary of the device stability for reported perovskite/perovskite/silicon triple-junction cells

Year	Stability performance	Refs
2018	n.a	[32]
2022	Observed a voltage drop after 0.5 h of *V*_OC_ tracking under continuous 1-sun illumination	[73]
2023	Retained 98.8% of its initial PCE after 0.16 h of MPP tracking under continuous 1-sun illumination	[74]
2023	Observed no PCE degradation after 0.1 h of MPP tracking under continuous 1-sun illumination	[81]
2024	Retained 86.8% of its initial PCE after 100 h of MPP tracking under continuous 1-sun illumination	[76]
2024	Retained 88.5% of its initial PCE after 1,000 h of aging at 85 °C in an N₂-filled glovebox	[77]
2024	Retained 96.6% of its initial PCE after 1,081 h of aging at 85 °C in an N₂-filled glovebox Retained 96% of its initial PCE after 11 h of MPP tracking under continuous 1-sun illumination at 50 °C	[75]
2024	Retained 80% of its initial PCE after 300 h of MPP tracking under continuous 1-sun illumination at 60 °C and 50% RH Retained 96% its initial PCE after 700 h of exposure to 65 °C and 85% RH in dark conditions	[33]
2024	Retained 93% of its initial PCE after 4.66 h of MPP tracking under continuous 1-sun illumination in ambient conditions	[82]
2025	Retained 95% of its initial PCE after 13 h at fixed voltage (V_MPP_) under continuous 1-sun illumination in ambient conditions	[83]
2025	Stable for 0.17 h of V_MPP_ tracking under continuous 1-sun illumination	[84]

It is worth to note that there is significant variability in the stability testing conditions reported in these literatures, which complicates direct comparisons between results and hinders a clear understanding of degradation mechanisms in devices. In response to this issue, the International Summit on Organic PV Stability (ISOS) protocols and International Electrotechnical Commission (IEC) standards established in 2018 have provided a foundation for standardized stability assessments of perovskite photovoltaic devices. While compliance with IEC standards is considered a baseline requirement, achieving commercial viability demands more rigorous testing protocols [177]. These include extended testing durations, stricter performance criteria, and intermittent degradation monitoring throughout the test period, as recommended by ISOS. On the other hand, although accelerated aging tests in above protocols are intended to simulate the effects of long-term outdoor operation, insights from the Si PV industry indicate that these protocols often fail to fully capture the complexities of real-world degradation. Currently, there is a lack of published field performance data for PSTJSCs, likely due to the absence of mature and reliable encapsulation strategies tailored for these devices. Encapsulation plays a pivotal role in long-term stability, with material selection being particularly constrained by the thermal sensitivity of perovskite layers. Some reported stability studies of PSTJSCs utilize a basic glass-to-glass edge sealing configuration, which may offer inadequate protection without the addition of a full-area external encapsulant. Therefore, further research into advanced encapsulation materials and techniques is warranted. Promising candidates include polyolefin elastomer (POE), thermoplastic polyurethane (TPU), and commercial multilayer lamination approaches, all of which merit systematic evaluation for compatibility with PSTJSC requirements [178–181].

## Conclusion

In conclusion, this review emphasizes the significant potential of PSTJSCs in advancing next-generation solar energy technologies. The perovskite/perovskite/silicon triple-junction architecture strikes an optimal balance between achieving high PCE and managing device complexity, positioning it as a transformative solution for the PV industry. The evolution of PSTJSCs has seen remarkable progress, with PCEs surpassing 27%, driven by innovations in light management, device structure, and perovskite composition. Despite these achievements, key challenges remain. The development of triple-junction solar cells has been limited number of studies and several practical challenges. These include difficulties in achieving current matching among the three subcells, a shortage of stable and efficient intermediate bandgap materials, and increased fabrication complexity and cost. Moreover, current mismatch issues persist due to the limited tunability of medium-bandgap perovskites, while wide-bandgap perovskites often suffer from high *V*_OC_ deficits, undermining their PCE. The instability of PSTJSCs suffering from phase segregation under operational conditions also continues to be a pressing concern.

Herein, in the context of prompting the PSTJSCs to higher PCE and achieving long-term stability, we outline feasible directions in the field for future research.

1. To achieve higher-efficiency PSTJSCs through triple-junction current matching, the foremost challenge lies in developing a stable moderate-bandgap (~ 1.43 eV) perovskite absorber layer that addresses instability caused by low-dose Sn doping, particularly resolving the degradation issues in Sn-containing perovskites induced by subsequent fabrication processes such as ALD [182]. This requires synergistic material engineering to enhance structural robustness against Sn oxidation while optimizing ALD parameters to minimize chemical stresses on the Sn–Pb perovskite layer.
2. The crystallization and nucleation mechanisms of perovskite on planar silicon versus textured silicon substrates differ significantly, leading to variations in the quality of the light-absorbing layers. Consequently, one of critical steps involves optimizing the crystallization control of top and middle perovskite subcells on textured silicon to achieve higher-quality perovskite light-absorbing layers for PSTJSCs.
3. To achieve highly stable PSTJSCs, in addition to enhancing the intrinsic stability of moderate-bandgap Sn–Pb perovskite, the photostability of Br-rich wide-bandgap perovskite top subcells emerge as another critical determinant of overall device durability. Notably, perovskite quantum dots (QDs), leveraging their unique quantum confinement effects and nanoscale structural characteristics, inherently circumvent phase segregation issues prevalent in thin-film counterparts [183]. These attributes position them as promising candidates for constructing photostable PSTJSCs. Systematic integration of such QD-based architecture into tandem configurations warrants in-depth investigation to unlock their full stability advantages.
4. To date, reported triple-junction solar cells have been focused on areas around 1 cm^2^, with no literature on large-area PSTJSCs. Enhancing their industrial potential requires scalable fabrication strategies, including scalable perovskite layer deposition and high performance interconnecting layers, to ensure compatibility with manufacturing-scale processes.
5. Although there have been no reports on quadruple-junction silicon-based perovskite tandem solar cells to date, it is foreseeable that, with continued advancements in the efficiency and stability of PSTJSCs, quadruple-junction will become a research hotspot in the near future due to higher theoretical efficiency.

So far, although PSTJSCs are currently less efficient than their perovskite–silicon tandem counterparts and involve more complex fabrication processes, they remain an attractive long-term strategy. The addition of a third junction enables better spectral splitting, higher theoretical efficiency limits exceeding 50%, and reduced sensitivity to current mismatch under varying illumination. As device integration and material stability improve, PSTJSCs hold strong promise for pushing photovoltaic performance beyond the limitations of current tandem technologies.

## References

[1] X. Peng, L. Sun, K. Feng, H. Zhong, J. Liang et al., Extent of global decarbonization of the power sector through energy policies and governance capacity. Commun. Earth Environ. **5**, 321 (2024). 10.1038/s43247-024-01494-5

[2] Y. Zhou, Worldwide carbon neutrality transition? Energy efficiency, renewable, carbon trading and advanced energy policies. Energy Rev. **2**(2), 100026 (2023). 10.1016/j.enrev.2023.100026

[3] L. Kruitwagen, K.T. Story, J. Friedrich, L. Byers, S. Skillman et al., A global inventory of photovoltaic solar energy generating units. Nature **598**(7882), 604–610 (2021). 10.1038/s41586-021-03957-7 34707304 10.1038/s41586-021-03957-7

[4] *International Technology Roadmap for Photovoltaic* (ITRPV) 14th edition. (2023). https://www.vdma.eu/en-GB/international-technology-roadmap-photovoltaic

[5] J.P. Helveston, G. He, M.R. Davidson, Quantifying the cost savings of global solar photovoltaic supply chains. Nature **612**(7938), 83–87 (2022). 10.1038/s41586-022-05316-6 36289345 10.1038/s41586-022-05316-6

[6] S. Chu, A. Majumdar, Opportunities and challenges for a sustainable energy future. Nature **488**(7411), 294–303 (2012). 10.1038/nature11475 22895334 10.1038/nature11475

[7] Longi sets new world-record for silicon solar cell efficiency, launching 2nd generation ultra-efficient bc-based module (2024) https://www.longi.com/en/news/longi-hi-mo9-bc-world-record

[8] W. Shockley, The shockley-queisser limit. J. Appl. Phys. **32**(3), 510–519 (1961). 10.1063/1.1736034

[9] R. Wang, T. Huang, J. Xue, J. Tong, K. Zhu et al., Prospects for metal halide perovskite-based tandem solar cells. Nat. Photonics **15**(6), 411–425 (2021). 10.1038/s41566-021-00809-8

[10] M. Heydarian, M. Heydarian, P. Schygulla, S.K. Reichmuth, A.J. Bett et al., Recent progress in monolithic two-terminal perovskite-based triple-junction solar cells. Energy Environ. Sci. **17**(5), 1781–1818 (2024). 10.1039/d3ee02822d

[11] M. Yamaguchi, F. Dimroth, J.F. Geisz, N.J. Ekins-Daukes, Multi-junction solar cells paving the way for super high-efficiency. J. Appl. Phys. **129**(24), 240901 (2021). 10.1063/5.0048653

[12] A.W.Y. Ho-Baillie, J. Zheng, M.A. Mahmud, F.-J. Ma, D.R. McKenzie et al., Recent progress and future prospects of perovskite tandem solar cells. Appl. Phys. Rev. **8**(4), 041307 (2021). 10.1063/5.0061483

[13] F. Guo, N. Li, F.W. Fecher, N. Gasparini, C.O.R. Quiroz et al., A generic concept to overcome bandgap limitations for designing highly efficient multi-junction photovoltaic cells. Nat. Commun. **6**, 7730 (2015). 10.1038/ncomms8730 26177808 10.1038/ncomms8730 PMC4518253

[14] D. König, K. Casalenuovo, Y. Takeda, G. Conibeer, J.F. Guillemoles et al., Hot carrier solar cells: Principles, materials and design. Phys. E Low Dimension. Syst. Nanostruct. **42**(10), 2862–2866 (2010). 10.1016/j.physe.2009.12.032

[15] S.P. Philipps, A.W. Bett, III-V Multi-junction solar cells and concentrating photovoltaic (CPV) systems. Adv. Opt. Technol. **3**(5–6), 469–478 (2014). 10.1515/aot-2014-0051

[16] M. Anaya, G. Lozano, M.E. Calvo, H. Míguez, ABX3 perovskites for tandem solar cells. Joule **1**(4), 769–793 (2017). 10.1016/j.joule.2017.09.017

[17] K. Wang, Z. Jin, L. Liang, H. Bian, D. Bai et al., All-inorganic cesium lead iodide perovskite solar cells with stabilized efficiency beyond 15. Nat. Commun. **9**(1), 4544 (2018). 10.1038/s41467-018-06915-6 30382108 10.1038/s41467-018-06915-6 PMC6208436

[18] H. Shen, D. Walter, Y. Wu, K.C. Fong, D.A. Jacobs et al., Monolithic perovskite/Si tandem solar cells: pathways to over 30% efficiency. Adv. Energy Mater. **10**(13), 1902840 (2020). 10.1002/aenm.201902840

[19] M. Yamaguchi, III–V compound multi-junction solar cells: present and future. Sol. Energy Mater. Sol. Cells **75**(1–2), 261–269 (2003). 10.1016/S0927-0248(02)00168-X

[20] S. Essig, C. Allebé, T. Remo, J.F. Geisz, M.A. Steiner et al., Raising the one-Sun conversion efficiency of III–V/Si solar cells to 32.8% for two junctions and 35.9% for three junctions. Nat. Energy **2**(9), 17144 (2017). 10.1038/nenergy.2017.144

[21] Martin A. Green, Ewan D. Dunlop, M. Yoshita, N. Kopidakis, K. Bothe et al., Solar cell efficiency tables (version 65). Prog. Photovoltaics **33**(1), 3–15 (2024). 10.1002/pip.3867

[22] K. Sasaki, T. Agui, K. Nakaido, N. Takahashi, R. Onitsuka et al., Development of InGaP/GaAs/InGaAs inverted triple junction concentrator solar cells. 9TH International Conference on Concentrator Photovoltaic Systems: CPV-9 Miyazaki, Japan. AIP, pp. 22–25 (2013). 10.1063/1.4822190

[23] P.T. Chiu, D.C. Law, R.L. Woo, S.B. Singer, D. Bhusari et al., 35.8% space and 38.8% terrestrial 5J direct bonded cells. 2014 IEEE 40th Photovoltaic Specialist Conference (PVSC). June 8–13, 2014, Denver, CO, USA. IEEE, pp. 0011–0013 (2014)

[24] J.F. Geisz, R.M. France, K.L. Schulte, M.A. Steiner, A.G. Norman et al., Six-junction III–V solar cells with 47.1% conversion efficiency under 143 Suns concentration. Nat. Energy **5**(4), 326–335 (2020). 10.1038/s41560-020-0598-5

[25] J.M. Raya-Armenta, N. Bazmohammadi, J.C. Vasquez, J.M. Guerrero, A short review of radiation-induced degradation of III–V photovoltaic cells for space applications. Sol. Energy Mater. Sol. Cells **233**, 111379 (2021). 10.1016/j.solmat.2021.111379

[26] H. Cotal, C. Fetzer, J. Boisvert, G. Kinsey, R. King et al., III–V multijunction solar cells for concentrating photovoltaics. Energy Environ. Sci. **2**(2), 174–192 (2009). 10.1039/b809257e

[27] H. Li, W. Zhang, Perovskite tandem solar cells: from fundamentals to commercial deployment. Chem. Rev. **120**(18), 9835–9950 (2020). 10.1021/acs.chemrev.9b00780 32786417 10.1021/acs.chemrev.9b00780

[28] P. Yan, D. Yang, H. Wang, S. Yang, Z. Ge, Recent advances in dopant-free organic hole-transporting materials for efficient, stable and low-cost perovskite solar cells. Energy Environ. Sci. **15**(9), 3630–3669 (2022). 10.1039/d2ee01256a

[29] P. Chen, Y. Xiao, S. Li, X. Jia, D. Luo et al., The promise and challenges of inverted perovskite solar cells. Chem. Rev. **124**(19), 10623–10700 (2024). 10.1021/acs.chemrev.4c00073 39207782 10.1021/acs.chemrev.4c00073

[30] S. Teale, M. Degani, B. Chen, E.H. Sargent, G. Grancini, Molecular cation and low-dimensional perovskite surface passivation in perovskite solar cells. Nat. Energy **9**(7), 779–792 (2024). 10.1038/s41560-024-01529-3

[31] NREL. Best research-cell efficiency chart (2025)

[32] J. Werner, F. Sahli, F. Fu, J.J. Diaz Leon, A. Walter et al., Perovskite/perovskite/silicon monolithic triple-junction solar cells with a fully textured design. ACS Energy Lett. **3**(9), 2052–2058 (2018). 10.1021/acsenergylett.8b01165

[33] S. Liu, Y. Lu, C. Yu, J. Li, R. Luo et al., Triple-junction solar cells with cyanate in ultrawide-bandgap perovskites. Nature **628**(8007), 306–312 (2024). 10.1038/s41586-024-07226-1 38438067 10.1038/s41586-024-07226-1

[34] F.H. Isikgor, T. Maksudov, X. Chang, B. Adilbekova, Z. Ling et al., Monolithic perovskite–perovskite–organic triple-junction solar cells with a voltage output exceeding 3 V. ACS Energy Lett. **7**(12), 4469–4471 (2022). 10.1021/acsenergylett.2c02340

[35] S. Hu, J. Wang, P. Zhao, J. Pascual, J. Wang et al., Steering perovskite precursor solutions for multijunction photovoltaics. Nature **639**(8053), 93–101 (2025). 10.1038/s41586-024-08546-y 39715627 10.1038/s41586-024-08546-y PMC11882461

[36] J. Wang, L. Zeng, D. Zhang, A. Maxwell, H. Chen et al., Halide homogenization for low energy loss in 2-eV-bandgap perovskites and increased efficiency in all-perovskite triple-junction solar cells. Nat. Energy **9**(1), 70–80 (2024). 10.1038/s41560-023-01406-5

[37] J. Liu, Y. He, L. Ding, H. Zhang, Q. Li et al., Perovskite/silicon tandem solar cells with bilayer interface passivation. Nature **635**(8039), 596–603 (2024). 10.1038/s41586-024-07997-7 39236747 10.1038/s41586-024-07997-7

[38] J. Liu, B. Shi, Q. Xu, Y. Li, Y. Li et al., Textured perovskite/silicon tandem solar cells achieving over 30% efficiency promoted by 4-fluorobenzylamine hydroiodide. Nano-Micro Lett. **16**(1), 189 (2024). 10.1007/s40820-024-01406-4 10.1007/s40820-024-01406-4 PMC11065830 38698120

[39] C. Kan, P. Hang, S. Wang, B. Li, X. Yu et al., Efficient and stable perovskite-silicon tandem solar cells with copper thiocyanate-embedded perovskite on textured silicon. Nat. Photonics **19**(1), 63–70 (2024). 10.1038/s41566-024-01561-5

[40] X. Li, Z. Ying, S. Li, L. Chen, M. Zhang et al., Top-down dual-interface carrier management for highly efficient and stable perovskite/silicon tandem solar cells. Nano-Micro Lett. **17**(1), 141 (2025). 10.1007/s40820-024-01631-x 10.1007/s40820-024-01631-x PMC11813841 39932612

[41] C. Li, Y. Chen, Y. Li, Z. Zhang, J. Yang et al., Achieving 32% efficiency in perovskite/silicon tandem solar cells with bidentate-anchored superwetting self-assembled molecular layers. Angew. Chem. Int. Ed. **64**(23), e202502730 (2025). 10.1002/anie.202502730 10.1002/anie.202502730 40171765

[42] C. Sun, N. University, et al., Wide-bandgap perovskite and perovskite/silicon tandem solar cells through strong hydrogen bonding interaction. ACS Energy Lett. **10**(5), 2171–2179 (2025). 10.1021/acsenergylett.5c00147

[43] L.C. Hirst, N.J. Ekins-Daukes, Fundamental losses in solar cells. Prog. Photovolt. Res. Appl. **19**(3), 286–293 (2011). 10.1002/pip.1024

[44] G.E. Eperon, M.T. Hörantner, H.J. Snaith, Metal halide perovskite tandem and multiple-junction photovoltaics. Nat. Rev. Chem. **1**(12), 0095 (2017). 10.1038/s41570-017-0095

[45] M.T. Hörantner, T. Leijtens, M.E. Ziffer, G.E. Eperon, M.G. Christoforo et al., The potential of multijunction perovskite solar cells. ACS Energy Lett. **2**(10), 2506–2513 (2017). 10.1021/acsenergylett.7b00647

[46] U. Rau, Reciprocity relation between photovoltaic quantum efficiency and electroluminescent emission of solar cells. Phys. Rev. B **76**(8), 085303 (2007). 10.1103/physrevb.76.085303

[47] S.R. Kurtz, P. Faine, J.M. Olson, Modeling of two-junction, series-connected tandem solar cells using top-cell thickness as an adjustable parameter. J. Appl. Phys. **68**(4), 1890–1895 (1990). 10.1063/1.347177

[48] F. Staub, I. Anusca, D.C. Lupascu, U. Rau, T. Kirchartz, Effect of reabsorption and photon recycling on photoluminescence spectra and transients in lead-halide perovskite crystals. J. Phys. Mater. **3**(2), 025003 (2020). 10.1088/2515-7639/ab6fd0

[49] A. Pusch, P. Pearce, N.J. Ekins-Daukes, Analytical expressions for the efficiency limits of radiatively coupled tandem solar cells. IEEE J. Photovolt. **9**(3), 679–687 (2019). 10.1109/JPHOTOV.2019.2903180

[50] M.A. Green, A. Ho-Baillie, H.J. Snaith, The emergence of perovskite solar cells. Nat. Photonics **8**(7), 506–514 (2014). 10.1038/nphoton.2014.134

[51] I. Borriello, G. Cantele, D. Ninno, Ab initio investigation of hybrid organic-inorganic perovskites based on tin halides. Phys. Rev. B **77**(23), 235214 (2008). 10.1103/PhysRevB.77.235214

[52] F.C. Hanusch, E. Wiesenmayer, E. Mankel, A. Binek, P. Angloher et al., Efficient planar heterojunction perovskite solar cells based on formamidinium lead bromide. J. Phys. Chem. Lett. **5**(16), 2791–2795 (2014). 10.1021/jz501237m 26278080 10.1021/jz501237m

[53] T. Jesper Jacobsson, J.-P. Correa-Baena, M. Pazoki, M. Saliba, K. Schenk et al., Exploration of the compositional space for mixed lead halogen perovskites for high efficiency solar cells. Energy Environ. Sci. **9**(5), 1706–1724 (2016). 10.1039/c6ee00030d

[54] J. Berry, T. Buonassisi, D.A. Egger, G. Hodes, L. Kronik et al., Hybrid organic–inorganic perovskites (HOIPs): opportunities and challenges. Adv. Mater. **27**(35), 5102–5112 (2015). 10.1002/adma.201502294 26223962 10.1002/adma.201502294

[55] J.L. Knutson, J.D. Martin, D.B. Mitzi, Tuning the band gap in hybrid tin iodide perovskite semiconductors using structural templating. Inorg. Chem. **44**(13), 4699–4705 (2005). 10.1021/ic050244q 15962978 10.1021/ic050244q

[56] P. Qin, A.L. Domanski, A.K. Chandiran, R. Berger, H.J. Butt et al., Yttrium-substituted nanocrystalline TiO₂ photoanodes for perovskite based heterojunction solar cells. Nanoscale **6**(3), 1508–1514 (2014). 10.1039/c3nr05884k 24322660 10.1039/c3nr05884k

[57] N.K. Noel, S.D. Stranks, A. Abate, C. Wehrenfennig, S. Guarnera et al., Lead-free organic–inorganic tin halide perovskites for photovoltaic applications. Energy Environ. Sci. **7**(9), 3061–3068 (2014). 10.1039/c4ee01076k

[58] P. Gao, M. Grätzel, M.K. Nazeeruddin, Organohalide lead perovskites for photovoltaic applications. Energy Environ. Sci. **7**(8), 2448–2463 (2014). 10.1039/c4ee00942h

[59] E.T. Hoke, D.J. Slotcavage, E.R. Dohner, A.R. Bowring, H.I. Karunadasa et al., Reversible photo-induced trap formation in mixed-halide hybrid perovskites for photovoltaics. Chem. Sci. **6**(1), 613–617 (2015). 10.1039/c4sc03141e 28706629 10.1039/c4sc03141e PMC5491962

[60] G.E. Eperon, S.D. Stranks, C. Menelaou, M.B. Johnston, L.M. Herz et al., Formamidinium lead trihalide: a broadly tunable perovskite for efficient planar heterojunction solar cells. Energy Environ. Sci. **7**(3), 982 (2014). 10.1039/c3ee43822h

[61] M.R. Filip, G.E. Eperon, H.J. Snaith, F. Giustino, Steric engineering of metal-halide perovskites with tunable optical band gaps. Nat. Commun. **5**, 5757 (2014). 10.1038/ncomms6757 25502506 10.1038/ncomms6757

[62] E. Mosconi, A. Amat, M.K. Nazeeruddin, M. Grätzel, F. De Angelis, First-principles modeling of mixed halide organometal perovskites for photovoltaic applications. J. Phys. Chem. C **117**(27), 13902–13913 (2013). 10.1021/jp4048659

[63] J. Im, C.C. Stoumpos, H. Jin, A.J. Freeman, M.G. Kanatzidis, Antagonism between spin–orbit coupling and steric effects causes anomalous band gap evolution in the perovskite photovoltaic materials CH_3_NH_3_Sn_1–__*x*_Pb_*x*_I_3_. J. Phys. Chem. Lett. **6**(17), 3503–3509 (2015). 10.1021/acs.jpclett.5b01738 27120685 10.1021/acs.jpclett.5b01738

[64] F. Hao, C.C. Stoumpos, D.H. Cao, R.P.H. Chang, M.G. Kanatzidis, Lead-free solid-state organic–inorganic halide perovskite solar cells. Nat. Photonics **8**(6), 489–494 (2014). 10.1038/nphoton.2014.82

[65] J. Lacombe, O. Sergeev, K. Chakanga, K. von Maydell, C. Agert, Three dimensional optical modeling of amorphous silicon thin film solar cells using the finite-difference time-domain method including real randomly surface topographies. J. Appl. Phys. **110**(2), 023102 (2011). 10.1063/1.3610516

[66] E. Centurioni, Generalized matrix method for calculation of internal light energy flux in mixed coherent and incoherent multilayers. Appl. Opt. **44**(35), 7532–7539 (2005). 10.1364/ao.44.007532 16363777 10.1364/ao.44.007532

[67] M.T. Hörantner, H.J. Snaith, Predicting and optimising the energy yield of perovskite-on-silicon tandem solar cells under real world conditions. Energy Environ. Sci. **10**(9), 1983–1993 (2017). 10.1039/c7ee01232b

[68] K. Jäger, J. Sutter, M. Hammerschmidt, P.-I. Schneider, C. Becker, Prospects of light management in perovskite/silicon tandem solar cells. Nanophotonics **10**(8), 1991–2000 (2021). 10.1515/nanoph-2020-0674

[69] S. Zandi, M. Razaghi, Finite element simulation of perovskite solar cell: a study on efficiency improvement based on structural and material modification. Sol. Energy **179**, 298–306 (2019). 10.1016/j.solener.2018.12.032

[70] M.I. Hossain, W. Qarony, S. Ma, L. Zeng, D. Knipp et al., Perovskite/silicon tandem solar cells: from detailed balance limit calculations to photon management. Nano-Micro Lett. **11**(1), 58 (2019). 10.1007/s40820-019-0287-8 10.1007/s40820-019-0287-8 PMC7770688 34138021

[71] L. Ba, H. Liu, W. Shen, Perovskite/c-Si tandem solar cells with realistic inverted architecture: Achieving high efficiency by optical optimization. Prog. Photovolt. Res. Appl. **26**(11), 924–933 (2018). 10.1002/pip.3037

[72] J. Li, P. Monk, D. Weile, Time domain integral equation methods in computational electromagnetism. Computational Electromagnetism. Springer International Publishing, (2015), pp 111–189 10.1007/978-3-319-19306-9_3

[73] J. Zheng, G. Wang, W. Duan, M.A. Mahmud, H. Yi et al., Monolithic perovskite–perovskite–silicon triple-junction tandem solar cell with an efficiency of over 20%. ACS Energy Lett. **7**(9), 3003–3005 (2022). 10.1021/acsenergylett.2c01556

[74] Y.J. Choi, S.Y. Lim, J.H. Park, S.G. Ji, J.Y. Kim, Atomic layer deposition-free monolithic perovskite/perovskite/silicon triple-junction solar cells. ACS Energy Lett. **8**(7), 3141–3146 (2023). 10.1021/acsenergylett.3c00919

[75] H. Hu, S.X. An, Y. Li, S. Orooji, R. Singh et al., Triple-junction perovskite–perovskite–silicon solar cells with power conversion efficiency of 24.4%. Energy Environ. Sci. **17**(8), 2800–2814 (2024). 10.1039/d3ee03687a 10.1039/d3ee03687a PMC11036531 38659971

[76] F. Li, D. Wu, L. Shang, R. Xia, H. Zhang et al., Highly efficient monolithic perovskite/perovskite/silicon triple-junction solar cells. Adv. Mater. **36**(16), e2311595 (2024). 10.1002/adma.202311595 38190828 10.1002/adma.202311595

[77] F. Xu, E. Aydin, J. Liu, E. Ugur, G.T. Harrison et al., Monolithic perovskite/perovskite/silicon triple-junction solar cells with cation double displacement enabled 2.0 eV perovskites. Joule **8**(1), 224–240 (2024). 10.1016/j.joule.2023.11.018

[78] Y. He, Z. Tang, B. He, C. Han, L. Ding et al., Composition engineering of perovskite absorber assisted efficient textured monolithic perovskite/silicon heterojunction tandem solar cells. RSC Adv. **13**(12), 7886–7896 (2023). 10.1039/d2ra05481g 36909745 10.1039/d2ra05481g PMC9996628

[79] Y. Li, B. Shi, Q. Xu, L. Yan, N. Ren et al., CsCl induced efficient fully-textured perovskite/crystalline silicon tandem solar cell. Nano Energy **122**, 109285 (2024). 10.1016/j.nanoen.2024.109285

[80] T.C. Yang, P. Fiala, Q. Jeangros, C. Ballif, High-bandgap perovskite materials for multijunction solar cells. Joule **2**(8), 1421–1436 (2018). 10.1016/j.joule.2018.05.008

[81] M. Heydarian, M. Heydarian, A.J. Bett, M. Bivour, F. Schindler et al., Monolithic two-terminal perovskite/perovskite/silicon triple-junction solar cells with open circuit voltage >2.8 V. ACS Energy Lett. **8**(10), 4186–4192 (2023). 10.1021/acsenergylett.3c01391 10.1021/acsenergylett.3c01391 PMC10580312 37854048

[82] T. Ye, L. Qiao, T. Wang, P. Wang, L. Zhang et al., Molecular synergistic effect for high efficiency monolithic perovskite/perovskite/silicon triple-junction tandem solar cells. Adv. Energy Mater. **14**(44), 2402491 (2024). 10.1002/aenm.202402491

[83] M. Heydarian, A. Shaji, O. Fischer, M. Günthel, O. Karalis et al., Minimizing open-circuit voltage losses in perovskite/perovskite/silicon triple-junction solar cell with optimized top cell. Sol. RRL **9**(3), 2400645 (2025). 10.1002/solr.202400645

[84] Y. Shao, S. Wang, T. Luo, C. Xu, J. Liu et al., Multi-functional interface engineering for monolithic perovskite/perovskite/crystalline silicon triple-junction tandem solar cells. Chemsuschem **18**(11), e202402680 (2025). 10.1002/cssc.202402680 39891546 10.1002/cssc.202402680

[85] J. Liu, M. De Bastiani, E. Aydin, G.T. Harrison, Y. Gao et al., Efficient and stable perovskite-silicon tandem solar cells through contact displacement by MgF _*x*_. Science **377**(6603), 302–306 (2022). 10.1126/science.abn8910 35737811 10.1126/science.abn8910

[86] Y. Chen, N. Yang, G. Zheng, F. Pei, W. Zhou et al., Nuclei engineering for even halide distribution in stable perovskite/silicon tandem solar cells. Science **385**(6708), 554–560 (2024). 10.1126/science.ado9104 39088618 10.1126/science.ado9104

[87] G. Wang, J. Zheng, W. Duan, J. Yang, M.A. Mahmud et al., Molecular engineering of hole-selective layer for high band gap perovskites for highly efficient and stable perovskite-silicon tandem solar cells. Joule **7**(11), 2583–2594 (2023). 10.1016/j.joule.2023.09.007

[88] J. Liu, E. Aydin, J. Yin, M. De Bastiani, F.H. Isikgor et al., 28.2%-efficient, outdoor-stable perovskite/silicon tandem solar cell. Joule **5**(12), 3169–3186 (2021). 10.1016/j.joule.2021.11.003

[89] J. Zheng, Z. Ying, Z. Yang, Z. Lin, H. Wei et al., Polycrystalline silicon tunnelling recombination layers for high-efficiency perovskite/tunnel oxide passivating contact tandem solar cells. Nat. Energy **8**(11), 1250–1261 (2023). 10.1038/s41560-023-01382-w

[90] L. Li, Y. Wang, X. Wang, R. Lin, X. Luo et al., Flexible all-perovskite tandem solar cells approaching 25% efficiency with molecule-bridged hole-selective contact. Nat. Energy **7**(8), 708–717 (2022). 10.1038/s41560-022-01045-2

[91] J. Zheng, W. Duan, Y. Guo, Z.C. Zhao, H. Yi et al., Efficient monolithic perovskite–Si tandem solar cells enabled by an ultra-thin indium tin oxide interlayer. Energy Environ. Sci. **16**(3), 1223–1233 (2023). 10.1039/d2ee04007g

[92] Z. Guo, A.K. Jena, G.M. Kim, T. Miyasaka, The high open-circuit voltage of perovskite solar cells: a review. Energy Environ. Sci. **15**(8), 3171–3222 (2022). 10.1039/d2ee00663d

[93] J. Hu, L. Yang, J. Zhang, A review on strategies to fabricate and stabilize phase-pure α-FAPbI_3_ perovskite solar cells. Sol. RRL **7**(13), 2300187 (2023). 10.1002/solr.202300187

[94] Z. Huang, Y. Bai, X. Huang, J. Li, Y. Wu et al., Anion-π interactions suppress phase impurities in FAPbI_3_ solar cells. Nature **623**(7987), 531–537 (2023). 10.1038/s41586-023-06637-w 37853122 10.1038/s41586-023-06637-w

[95] X. Jiang, X. Wang, X. Wu, S. Zhang, B. Liu et al., Strain regulation *via* pseudo halide-based ionic liquid toward efficient and stable *α*-FAPbI_3_ inverted perovskite solar cells. Adv. Energy Mater. **13**(23), 2300700 (2023). 10.1002/aenm.202300700

[96] Z. Zheng, S. Wang, Y. Hu, Y. Rong, A. Mei et al., Development of formamidinium lead iodide-based perovskite solar cells: efficiency and stability. Chem. Sci. **13**(8), 2167–2183 (2022). 10.1039/D1SC04769H 35310498 10.1039/d1sc04769h PMC8865136

[97] M. RaeisianAsl, S.F.K.S. Panahi, M. Jamaati, S.S. Tafreshi, A review on theoretical studies of structural and optoelectronic properties of FA-based perovskite materials with a focus on FAPbI_3_. Int. J. Energy Res. **46**(10), 13117–13151 (2022). 10.1002/er.8008

[98] G. Liu, L. Kong, J. Gong, W. Yang, H.-K. Mao et al., Pressure-induced bandgap optimization in lead-based perovskites with prolonged carrier lifetime and ambient retainability. Adv. Funct. Mater. **27**(3), 1604208 (2017). 10.1002/adfm.201604208

[99] E.I. Marchenko, S.A. Fateev, V.V. Korolev, V. Buchinskiy, N.N. Eremin et al., Structure-related bandgap of hybrid lead halide perovskites and close-packed APbX_3_ family of phases. J. Mater. Chem. C **10**(44), 16838–16846 (2022). 10.1039/d2tc03202c

[100] H. Min, M. Kim, S.U. Lee, H. Kim, G. Kim et al., Efficient, stable solar cells by using inherent bandgap of α-phase formamidinium lead iodide. Science **366**(6466), 749–753 (2019). 10.1126/science.aay7044 31699938 10.1126/science.aay7044

[101] G. Kim, H. Min, K.S. Lee, D.Y. Lee, S.M. Yoon et al., Impact of strain relaxation on performance of α-formamidinium lead iodide perovskite solar cells. Science **370**(6512), 108–112 (2020). 10.1126/science.abc4417 33004518 10.1126/science.abc4417

[102] H. Lu, Y. Liu, P. Ahlawat, A. Mishra, W.R. Tress et al., Vapor-assisted deposition of highly efficient, stable black-phase FAPbI_3_ perovskite solar cells. Science **370**(6512), eabb8985 (2020). 10.1126/science.abb8985 10.1126/science.abb8985 33004488

[103] J. Jeong, M. Kim, J. Seo, H. Lu, P. Ahlawat et al., Pseudo-halide anion engineering for α-FAPbI_3_ perovskite solar cells. Nature **592**(7854), 381–385 (2021). 10.1038/s41586-021-03406-5 33820983 10.1038/s41586-021-03406-5

[104] H. Chen, Y. Chen, T. Zhang, X. Liu, X. Wang et al., Advances to high-performance black-phase FAPbI3 perovskite for efficient and stable photovoltaics. Small Struct. **2**(5), 2000130 (2021). 10.1002/sstr.202000130

[105] K. Li, H. Zhou, Advances to stabilize photoactive phase of FAPbI_3_ perovskite. Chin. J. Chem. **41**(20), 2730–2745 (2023). 10.1002/cjoc.202300128

[106] Y. Huang, X. Lei, T. He, Y. Jiang, M. Yuan, Recent progress on formamidinium-dominated perovskite photovoltaics. Adv. Energy Mater. **12**(4), 2100690 (2022). 10.1002/aenm.202100690

[107] J. Cao, F. Yan, Recent progress in tin-based perovskite solar cells. Energy Environ. Sci. **14**(3), 1286–1325 (2021). 10.1039/d0ee04007j

[108] A. Yadegarifard, H. Lee, H.-J. Seok, I. Kim, B.-K. Ju et al., FA/Cs-based mixed Pb–Sn perovskite solar cells: a review of recent advances in stability and efficiency. Nano Energy **112**, 108481 (2023). 10.1016/j.nanoen.2023.108481

[109] W. Ke, C.C. Stoumpos, M.G. Kanatzidis, “Unleaded” perovskites: status quo and future prospects of tin-based perovskite solar cells. Adv. Mater. **31**(47), 1803230 (2019). 10.1002/adma.201803230 10.1002/adma.201803230 30276882

[110] S. Hu, J. Thiesbrummel, J. Pascual, M. Stolterfoht, A. Wakamiya et al., Narrow bandgap metal halide perovskites for all-perovskite tandem photovoltaics. Chem. Rev. **124**(7), 4079–4123 (2024). 10.1021/acs.chemrev.3c00667 38527274 10.1021/acs.chemrev.3c00667 PMC11009966

[111] F. Yang, K. Zhu, Advances in mixed tin-lead narrow-bandgap perovskites for single-junction and all-perovskite tandem solar cells. Adv. Mater. **36**(31), 2314341 (2024). 10.1002/adma.202314341 10.1002/adma.202314341 38779891

[112] Z. Yang, A. Rajagopal, A.K.Y. Jen, Ideal bandgap organic–inorganic hybrid perovskite solar cells. Adv. Mater. **29**(47), 1704418 (2017). 10.1002/adma.201704418 10.1002/adma.201704418 29134752

[113] T. AlZoubi, W.J. Kadhem, M. Al Gharram, G. Makhadmeh, M.A.O. Abdelfattah et al., Advanced optoelectronic modeling and optimization of HTL-free FASnI_3_/C60 perovskite solar cell architecture for superior performance. Nanomaterials **14**(12), 1062 (2024). 10.3390/nano14121062 10.3390/nano14121062 PMC11206542 38921938

[114] B.-B. Yu, Z. Chen, Y. Zhu, Y. Wang, B. Han et al., Heterogeneous 2D/3D tin-halides perovskite solar cells with certified conversion efficiency breaking 14%. Adv. Mater. **33**(36), 2102055 (2021). 10.1002/adma.202102055 10.1002/adma.202102055 34296476

[115] S. Lv, W. Gao, Y. Liu, H. Dong, N. Sun et al., Stability of Sn-Pb mixed organic–inorganic halide perovskite solar cells: Progress, challenges, and perspectives. J. Energy Chem. **65**, 371–404 (2022). 10.1016/j.jechem.2021.06.011

[116] A. Halder, K.-W. Yeom, N.-G. Park, Strategies toward suppression of Sn(II) oxidation for stable Sn–Pb perovskite solar cells. ACS Energy Lett. **8**(10), 4267–4277 (2023). 10.1021/acsenergylett.3c01610

[117] J. Liu, H. Yao, S. Wang, C. Wu, L. Ding et al., Origins and suppression of Sn(II)/Sn(IV) oxidation in tin halide perovskite solar cells. Adv. Energy Mater. **13**(23), 2300696 (2023). 10.1002/aenm.202300696

[118] H. Dong, C. Ran, W. Gao, N. Sun, X. Liu et al., Crystallization dynamics of Sn-based perovskite thin films: toward efficient and stable photovoltaic devices. Adv. Energy Mater. **12**(1), 2102213 (2022). 10.1002/aenm.202102213

[119] T.J. MacDonald, L. Lanzetta, X. Liang, D. Ding, S.A. Haque, Engineering stable lead-free tin halide perovskite solar cells: lessons from materials chemistry. Adv. Mater. **35**(25), 2206684 (2023). 10.1002/adma.202206684 10.1002/adma.202206684 36458662

[120] E.W. Diau, E. Jokar, M. Rameez, Strategies to improve performance and stability for tin-based perovskite solar cells. ACS Energy Lett. **4**(8), 1930–1937 (2019). 10.1021/acsenergylett.9b01179

[121] L. Chen, S. Fu, Y. Li, N. Sun, Y. Yan et al., On the durability of tin-containing perovskite solar cells. Adv. Sci. **11**(1), e2304811 (2024). 10.1002/advs.202304811 10.1002/advs.202304811 PMC10767427 37968252

[122] P. Li, X. Cao, J. Li, B. Jiao, X. Hou et al., Ligand engineering in tin-based perovskite solar cells. Nano-Micro Lett. **15**(1), 167 (2023). 10.1007/s40820-023-01143-0 10.1007/s40820-023-01143-0 PMC10317948 37395847

[123] M. Wright, B. Vicari Stefani, T.W. Jones, B. Hallam, A. Soeriyadi et al., Design considerations for the bottom cell in perovskite/silicon tandems: a terawatt scalability perspective. Energy Environ. Sci. **16**(10), 4164–4190 (2023). 10.1039/d3ee00952a

[124] G. Hashmi, M. Hasanuzzaman, M.K. Basher, M. Hoq, M.H. Rahman, Texturization of as-cut p-type monocrystalline silicon wafer using different wet chemical solutions. Appl. Phys. A Mater. Sci. Process. **124**(6), 415 (2018). 10.1007/s00339-018-1818-8

[125] F. Fu, J. Li, T.C. Yang, H. Liang, A. Faes et al., Monolithic perovskite-silicon tandem solar cells: from the lab to fab? Adv. Mater. **34**(24), 2106540 (2022). 10.1002/adma.202106540 10.1002/adma.202106540 35060205

[126] D.H. MacDonald, A. Cuevas, M.J. Kerr, C. Samundsett, D. Ruby et al., Texturing industrial multicrystalline silicon solar cells. Sol. Energy **76**(1–3), 277–283 (2004). 10.1016/j.solener.2003.08.019

[127] D.Z. Dimitrov, C.-H. Du, Crystalline silicon solar cells with micro/nano texture. Appl. Surf. Sci. **266**, 1–4 (2013). 10.1016/j.apsusc.2012.10.081

[128] X. Luo, H. Luo, H. Li, R. Xia, X. Zheng et al., Efficient perovskite/silicon tandem solar cells on industrially compatible textured silicon. Adv. Mater. **35**(9), e2207883 (2023). 10.1002/adma.202207883 36599055 10.1002/adma.202207883

[129] P. Tockhorn, J. Sutter, A. Cruz, P. Wagner, K. Jäger et al., Nano-optical designs for high-efficiency monolithic perovskite-silicon tandem solar cells. Nat. Nanotechnol. **17**(11), 1214–1221 (2022). 10.1038/s41565-022-01228-8 36280763 10.1038/s41565-022-01228-8 PMC9646483

[130] A. Harter, S. Mariotti, L. Korte, R. Schlatmann, S. Albrecht et al., Double-sided nano-textured surfaces for industry compatible high-performance silicon heterojunction and perovskite/silicon tandem solar cells. Prog. Photovolt. Res. Appl. **31**(8), 813–823 (2023). 10.1002/pip.3685

[131] A. Alasfour, Z.J. Yu, W. Weigand, D. Quispe, Z.C. Holman, Sub-micrometer random-pyramid texturing of silicon solar wafers with excellent surface passivation and low reflectance. Sol. Energy Mater. Sol. Cells **218**, 110761 (2020). 10.1016/j.solmat.2020.110761

[132] F. Sahli, J. Werner, B.A. Kamino, M. Bräuninger, R. Monnard et al., Fully textured monolithic perovskite/silicon tandem solar cells with 25.2% power conversion efficiency. Nat. Mater. **17**(9), 820–826 (2018). 10.1038/s41563-018-0115-4 10.1038/s41563-018-0115-4 29891887

[133] L. Mao, T. Yang, H. Zhang, J. Shi, Y. Hu et al., Fully textured, production-line compatible monolithic perovskite/silicon tandem solar cells approaching 29% efficiency. Adv. Mater. **34**(40), e2206193 (2022). 10.1002/adma.202206193 35985840 10.1002/adma.202206193

[134] F. Hou, X. Ren, H. Guo, X. Ning, Y. Wang et al., Monolithic perovskite/silicon tandem solar cells: a review of the present status and solutions toward commercial application. Nano Energy **124**, 109476 (2024). 10.1016/j.nanoen.2024.109476

[135] Y. Shi, J.J. Berry, F. Zhang, Perovskite/silicon tandem solar cells: insights and outlooks. ACS Energy Lett. **9**(3), 1305–1330 (2024). 10.1021/acsenergylett.4c00172

[136] Z. Wu, E. Bi, C. Li, L. Chen, Z. Song et al., Scalable two-step production of high-efficiency perovskite solar cells and modules. Sol. RRL **7**(1), 2200571 (2023). 10.1002/solr.202200571

[137] J.-H. Lee, B.S. Kim, J. Park, J.-W. Lee, K. Kim, Opportunities and challenges for perovskite solar cells based on vacuum thermal evaporation. Adv. Mater. Technol. **8**(20), 2200928 (2023). 10.1002/admt.202200928

[138] Z. Wang, M. Lyu, B.W. Zhang, M. Xiao, C. Zhang et al., Thermally evaporated metal halide perovskites and their analogues: film fabrication, applications and beyond. Small Meth. **9**(2), 2301633 (2025). 10.1002/smtd.202301633 10.1002/smtd.202301633 PMC11843433 38682581

[139] Y. Tong, A. Najar, L. Wang, L. Liu, M. Du et al., Wide-bandgap organic-inorganic lead halide perovskite solar cells. Adv. Sci. **9**(14), e2105085 (2022). 10.1002/advs.202105085 10.1002/advs.202105085 PMC9109050 35257511

[140] K. Suchan, T.J. Jacobsson, C. Rehermann, E.L. Unger, T. Kirchartz et al., Rationalizing performance losses of wide bandgap perovskite solar cells evident in data from the perovskite database. Adv. Energy Mater. **14**(5), 2303420 (2024). 10.1002/aenm.202303420

[141] M. Stolterfoht, C.M. Wolff, J.A. Márquez, S. Zhang, C.J. Hages et al., Visualization and suppression of interfacial recombination for high-efficiency large-area pin perovskite solar cells. Nat. Energy **3**(10), 847–854 (2018). 10.1038/s41560-018-0219-8

[142] O. Almora, G.C. Bazan, C.I. Cabrera, L.A. Castriotta, S. Erten-Ela et al., Device performance of emerging photovoltaic materials (version 5). Adv. Energy Mater. **15**(12), 2404386 (2024). 10.1002/aenm.202404386

[143] W. Yang, H. Long, X. Sha, J. Sun, Y. Zhao et al., Unlocking voltage potentials of mixed-halide perovskite solar cells *via* phase segregation suppression. Adv. Funct. Mater. **32**(12), 2110698 (2022). 10.1002/adfm.202110698

[144] A. Rajagopal, R.J. Stoddard, S.B. Jo, H.W. Hillhouse, A.K. Jen, Overcoming the photovoltage plateau in large bandgap perovskite photovoltaics. Nano Lett. **18**(6), 3985–3993 (2018). 10.1021/acs.nanolett.8b01480 29733214 10.1021/acs.nanolett.8b01480

[145] Y. An, N. Zhang, Z. Zeng, Y. Cai, W. Jiang et al., Optimizing crystallization in wide-bandgap mixed halide perovskites for high-efficiency solar cells. Adv. Mater. **36**(17), 2306568 (2024). 10.1002/adma.202306568 10.1002/adma.202306568 37677058

[146] Y.J. Ahn, H.J. Kim, I.J. Park, J.Y. Kim, Recent advances and opportunities in perovskite-based triple-junction tandem solar cells. Sustain. Energy Fuels **8**(23), 5352–5365 (2024). 10.1039/d4se01051e

[147] G.F. Samu, Á. Balog, F. De Angelis, D. Meggiolaro, P.V. Kamat et al., Electrochemical hole injection selectively expels iodide from mixed halide perovskite films. J. Am. Chem. Soc. **141**(27), 10812–10820 (2019). 10.1021/jacs.9b04568 31259546 10.1021/jacs.9b04568 PMC6624782

[148] G. Yang, Z. Ni, Z.J. Yu, B.W. Larson, Z. Yu et al., Defect engineering in wide-bandgap perovskites for efficient perovskite–silicon tandem solar cells. Nat. Photon. **16**(8), 588–594 (2022). 10.1038/s41566-022-01033-8

[149] Y. Zhou, I. Poli, D. Meggiolaro, F. De Angelis, A. Petrozza, Defect activity in metal halide perovskites with wide and narrow bandgap. Nat. Rev. Mater. **6**(11), 986–1002 (2021). 10.1038/s41578-021-00331-x

[150] T. Nie, Z. Fang, X. Ren, Y. Duan, S.F. Liu, Recent advances in wide-bandgap organic-inorganic halide perovskite solar cells and tandem application. Nano-Micro Lett. **15**(1), 70 (2023). 10.1007/s40820-023-01040-6 10.1007/s40820-023-01040-6 PMC10030759 36943501

[151] A.J. Ramadan, R.D.J. Oliver, M.B. Johnston, H.J. Snaith, Methylammonium-free wide-bandgap metal halide perovskites for tandem photovoltaics. Nat. Rev. Mater. **8**(12), 822–838 (2023). 10.1038/s41578-023-00610-9

[152] F. Xu, M. Zhang, Z. Li, X. Yang, R. Zhu, Challenges and perspectives toward future wide-bandgap mixed-halide perovskite photovoltaics. Adv. Energy Mater. **13**(13), 2203911 (2023). 10.1002/aenm.202203911

[153] Z. Cheng, M. Zhang, Y. Zhang, W. Qi, Z. Wang et al., Stable wide-bandgap perovskite solar cells for tandem applications. Nano Energy **127**, 109708 (2024). 10.1016/j.nanoen.2024.109708

[154] C.M. Wolff, P. Caprioglio, M. Stolterfoht, D. Neher, Nonradiative recombination in perovskite solar cells: the role of interfaces. Adv. Mater. **31**(52), e1902762 (2019). 10.1002/adma.201902762 31631441 10.1002/adma.201902762

[155] H. Chen, A. Maxwell, C. Li, S. Teale, B. Chen et al., Regulating surface potential maximizes voltage in all-perovskite tandems. Nature **613**(7945), 676–681 (2023). 10.1038/s41586-022-05541-z 36379225 10.1038/s41586-022-05541-z

[156] T. Li, J. Xu, R. Lin, S. Teale, H. Li et al., Inorganic wide-bandgap perovskite subcells with dipole bridge for all-perovskite tandems. Nat. Energy **8**(6), 610–620 (2023). 10.1038/s41560-023-01250-7

[157] N. Ahn, M. Choi, Towards long-term stable perovskite solar cells: degradation mechanisms and stabilization techniques. Adv. Sci. **11**(4), 2306110 (2024). 10.1002/advs.202306110 10.1002/advs.202306110 PMC10811515 37997198

[158] L. Duan, A. Uddin, Defects and stability of perovskite solar cells: a critical analysis. Mater. Chem. Front. **6**(4), 400–417 (2022). 10.1039/d1qm01250a

[159] T.A. Chowdhury, M.A. Bin Zafar, M. Sajjad-Ul Islam, M. Shahinuzzaman, M.A. Islam et al., Stability of perovskite solar cells: issues and prospects. RSC Adv. **13**(3), 1787–1810 (2023). 10.1039/d2ra05903g 10.1039/d2ra05903g PMC9828105 36712629

[160] G. Nazir, S.-Y. Lee, J.-H. Lee, A. Rehman, J.-K. Lee et al., Stabilization of perovskite solar cells: recent developments and future perspectives. Adv. Mater. **34**(50), 2204380 (2022). 10.1002/adma.202204380 10.1002/adma.202204380 36103603

[161] H. Zhang, L. Pfeifer, S.M. Zakeeruddin, J. Chu, M. Grätzel, Tailoring passivators for highly efficient and stable perovskite solar cells. Nat. Rev. Chem. **7**(9), 632–652 (2023). 10.1038/s41570-023-00510-0 37464018 10.1038/s41570-023-00510-0

[162] A.R. bin Mohd Yusoff, M. Vasilopoulou, D.G. Georgiadou, L.C. Palilis, A. Abate et al., Passivation and process engineering approaches of halide perovskite films for high efficiency and stability perovskite solar cells. Energy Environ. Sci. **14**(5), 2906–2953 (2021). 10.1039/d1ee00062d

[163] X. Ren, J. Wang, Y. Lin, Y. Wang, H. Xie et al., Mobile iodides capture for highly photolysis- and reverse-bias-stable perovskite solar cells. Nat. Mater. **23**(6), 810–817 (2024). 10.1038/s41563-024-01876-2 38684883 10.1038/s41563-024-01876-2

[164] H. Kim, S.M. Yoo, B. Ding, H. Kanda, N. Shibayama et al., Shallow-level defect passivation by 6H perovskite polytype for highly efficient and stable perovskite solar cells. Nat. Commun. **15**(1), 5632 (2024). 10.1038/s41467-024-50016-6 38965276 10.1038/s41467-024-50016-6 PMC11224362

[165] J. Zhou, L. Tan, Y. Liu, H. Li, X. Liu et al., Highly efficient and stable perovskite solar cells *via* a multifunctional hole transporting material. Joule **8**(6), 1691–1706 (2024). 10.1016/j.joule.2024.02.019

[166] P. Caprioglio, S. Caicedo-Dávila, T.C. Yang, C.M. Wolff, F. Peña-Camargo et al., Nano-emitting heterostructures violate optical reciprocity and enable efficient photoluminescence in halide-segregated methylammonium-free wide bandgap perovskites. ACS Energy Lett. **6**(2), 419–428 (2021). 10.1021/acsenergylett.0c02270

[167] L. Tian, J. Xue, R. Wang, Halide segregation in mixed halide perovskites: visualization and mechanisms. Electronics **11**(5), 700 (2022). 10.3390/electronics11050700

[168] K.A. Bush, K. Frohna, R. Prasanna, R.E. Beal, T. Leijtens et al., Compositional engineering for efficient wide band gap perovskites with improved stability to photoinduced phase segregation. ACS Energy Lett. **3**(2), 428–435 (2018). 10.1021/acsenergylett.7b01255

[169] S. Wu, M. Liu, A.K.Y. Jen, Prospects and challenges for perovskite-organic tandem solar cells. Joule **7**(3), 484–502 (2023). 10.1016/j.joule.2023.02.014

[170] D.J. Slotcavage, H.I. Karunadasa, M.D. McGehee, Light-induced phase segregation in halide-perovskite absorbers. ACS Energy Lett. **1**(6), 1199–1205 (2016). 10.1021/acsenergylett.6b00495

[171] L. Duan, D. Walter, N. Chang, J. Bullock, D. Kang et al., Stability challenges for the commercialization of perovskite–silicon tandem solar cells. Nat. Rev. Mater. **8**(4), 261–281 (2023). 10.1038/s41578-022-00521-1

[172] P. Chen, Y. Xiao, J. Hu, S. Li, D. Luo et al., Multifunctional ytterbium oxide buffer for perovskite solar cells. Nature **625**(7995), 516–522 (2024). 10.1038/s41586-023-06892-x 38233617 10.1038/s41586-023-06892-x

[173] S. Li, Y. Xiao, R. Su, W. Xu, D. Luo et al., Coherent growth of high-Miller-index facets enhances perovskite solar cells. Nature **635**(8040), 874–881 (2024). 10.1038/s41586-024-08159-5 39401515 10.1038/s41586-024-08159-5

[174] Z. Fang, T. Nie, S. Liu, J. Ding, Overcoming phase segregation in wide-bandgap perovskites: from progress to perspective. Adv. Funct. Mater. **34**(42), 2404402 (2024). 10.1002/adfm.202404402

[175] N. Porotnikova, D. Osinkin, Segregation and interdiffusion processes in perovskites: a review of recent advances. J. Mater. Chem. A **12**(5), 2620–2646 (2024). 10.1039/d3ta06708d

[176] Z. Cui, Q. Zhang, Y. Bai, Q. Chen, Issues of phase segregation in wide-bandgap perovskites. Mater. Chem. Front. **7**(10), 1896–1911 (2023). 10.1039/d2qm01341j

[177] M.V. Khenkin, E.A. Katz, A. Abate, G. Bardizza, J.J. Berry et al., Consensus statement for stability assessment and reporting for perovskite photovoltaics based on ISOS procedures. Nat. Energy **5**(1), 35–49 (2020). 10.1038/s41560-019-0529-5

[178] M. Kaltenbrunner, G. Adam, E.D. Głowacki, M. Drack, R. Schwödiauer et al., Flexible high power-per-weight perovskite solar cells with chromium oxide-metal contacts for improved stability in air. Nat. Mater. **14**(10), 1032–1039 (2015). 10.1038/nmat4388 26301766 10.1038/nmat4388

[179] B. Salhi, Y.S. Wudil, M.K. Hossain, A. Al-Ahmed, F.A. Al-Sulaiman, Review of recent developments and persistent challenges in stability of perovskite solar cells. Renew. Sustain. Energy Rev. **90**, 210–222 (2018). 10.1016/j.rser.2018.03.058

[180] Q. Lu, Z. Yang, X. Meng, Y. Yue, M.A. Ahmad et al., A review on encapsulation technology from organic light emitting diodes to organic and perovskite solar cells. Adv. Funct. Mater. **31**(23), 2100151 (2021). 10.1002/adfm.202100151

[181] J. Li, R. Xia, W. Qi, X. Zhou, J. Cheng et al., Encapsulation of perovskite solar cells for enhanced stability: Structures, materials and characterization. J. Power. Sources **485**, 229313 (2021). 10.1016/j.jpowsour.2020.229313

[182] A. Naikaew, P. Kumnorkaew, W. Wattanathana, K.Z. Swe, P. Pansa-Ngat et al., Investigation of double-layered Pb-Sn perovskite absorbers: formation, structure, band alignment, and stability. J. Phys. Chem. C **126**(3), 1623–1634 (2022). 10.1021/acs.jpcc.1c08811

[183] M. Hao, S. Ding, S. Gaznaghi, H. Cheng, L. Wang, Perovskite quantum dot solar cells: current status and future outlook. ACS Energy Lett. **9**(1), 308–322 (2024). 10.1021/acsenergylett.3c01983

